# *APOE4* genetic polymorphism results in impaired recovery in a repeated mild traumatic brain injury model and treatment with Bryostatin-1 improves outcomes

**DOI:** 10.1038/s41598-020-76849-x

**Published:** 2020-11-16

**Authors:** Anna O. Giarratana, Cynthia Zheng, Sahithi Reddi, Shavonne L. Teng, David Berger, Derek Adler, Patrick Sullivan, Smita Thakker-Varia, Janet Alder

**Affiliations:** 1 Department of Neuroscience and Cell Biology, Rutgers Robert Wood Johnson Medical School, 683 Hoes Lane West, Research Building 357A, Piscataway, NJ 08854-5635 USA; 2 Division of Geriatrics, Department of Medicine, Durham VAMC, GRECC, Duke University, Durham, NC USA; 3 Rutgers Molecular Imaging Center, 41 Gordon Road, Suite D, Room 365A, Piscataway, NJ 08854 USA

**Keywords:** Brain injuries, Diseases of the nervous system

## Abstract

After traumatic brain injury (TBI), some people have worse recovery than others. Single nucleotide polymorphisms (SNPs) in *Apolipoprotein E* (*APOE*) are known to increase risk for developing Alzheimer’s disease, however there is controversy from human and rodent studies as to whether ApoE4 is a risk factor for worse outcomes after brain trauma. To resolve these conflicting studies we have explored the effect of the human *APOE4* gene in a reproducible mouse model that mimics common human injuries. We have investigated cellular and behavioral outcomes in genetically engineered human *APOE* targeted replacement (TR) mice following repeated mild TBI (rmTBI) using a lateral fluid percussion injury model. Relative to injured *APOE3* TR mice, injured *APOE4* TR mice had more inflammation, neurodegeneration, apoptosis, p-tau, and activated microglia and less total brain-derived neurotrophic factor (BDNF) in the cortex and/or hippocampus at 1 and/or 21 days post-injury. We utilized a novel personalized approach to treating *APOE4* susceptible mice by administering Bryostatin-1, which improved cellular as well as motor and cognitive behavior outcomes at 1 DPI in the *APOE4* injured mice. This study demonstrates that *APOE4* is a risk factor for poor outcomes after rmTBI and highlights how personalized therapeutics can be a powerful treatment option.

## Introduction

Traumatic Brain Injury (TBI) results when there is an insult to the head which causes a disruption of normal brain function. Injuries can range in severity from mild to severe, with mild TBIs, commonly called concussions, being the most common form. In 2013, there were approximately 1.125 million reported mild TBIs in the United States, with costs of mild TBI reaching $117 billion each year^1^. Mild TBIs can actually cause very serious health problems, and it is estimated that around 15% of patients have symptoms that last longer than 3 months and develop into chronic disabilities^2^. This problem is further compounded when a person sustains a second mild TBI after the initial head injury. Athletes and military personnel, in particular, are at an increased risk for sustaining repeated mild TBI (rmTBI), which can have devastating cognition and psychological consequences^3,4^. The ability to recover after undergoing rmTBI is variable; some are able to recover quickly and have no obvious long-term problems, while others go on to develop lifelong complications^5,6^. One of the reasons for this differential response to rmTBI may be the genetic differences that exist in the population.

Apolipoprotein E (*APOE*) is a critical neuronal gene that contains two single nucleotide polymorphisms (SNPs) also referred to as rs429358 and rs7412, which affect the structure and function of the resultant protein^7^. The apoE protein is an important protein responsible for the transport and clearance of lipids and cholesterol in the brain. There are three isoforms: apoE2, apoE3, and apoE4. ApoE4 has an Arg at both the 112 and 158 position, apoE3 has a Cys at the 112 position, and apoE2 has a Cys at both the 112 and 158 positions^8^. *APOE3* is the most common human allele with an allelic frequency ranging from 53.6 to 89.8%, although there is considerable variation in allelic frequency across worldwide populations, with the ancestral *APOE4* allele having a frequency ranging from 5.2 to 40.7%^9^.

Previous studies in humans have investigated the effect of *APOE* genetic polymorphisms on health outcomes. Multiple studies have shown that carriers of the *APOE4* allele have the largest known genetic risk factor for the development of late onset Alzheimer’s disease^10–12^. Recently, there has been an interest in studying the effect of the *APOE4* allele on outcomes after brain injury^13^. Early studies looked retrospectively at athletes who had repeated concussions, and worse long-term outcomes were correlated with *APOE4* carrier status^14,15^. And while some human studies have shown that the *APOE4* allele is a risk factor for sustaining multiple concussions^16,17^, others have shown that having the *APOE4* allele is not a risk factor for a concussion^18^. Moreover, while an early meta-analysis showed that carriers of the *APOE4* allele had worse long-term outcomes^19^, a more recent meta-analysis showed that there was no association with *APOE4* status and poor cognitive outcomes after TBI^20^. Therefore, although there is some support in the literature for *APOE4* being a risk factor for worse outcomes after TBI in humans, there is no consensus among all the studies.

In order to elucidate the effect of *APOE* SNPs on recovery after TBI, a number of rodent studies have been performed, although these too remain controversial. In a closed head injury model, ApoE4 mice were twice as likely to die or have higher neurological severity scores compared to the ApoE3 mice^21^. However, there is no consensus on the pathology behind these results^22–24^. There are also conflicting reports on how the ApoE4 allele causes these detrimental effects, and the roles of age, type of injury, and other genetic alleles are not well studied^22,25–28^. Some researchers are examining the effect of the ApoE polymorphism after rmTBI. One study found that there was no effect of the ApoE4 genotype on recovery after repeated mild controlled cortical impact (CCI) injury^29^. Others found that after repeated blast injury, ApoE4 injured mice had higher levels of p-tau, which can result in tangles that contribute to cognitive decline, relative to ApoE3 injured mice^30^. Another recent study looked at the effect of ApoE4 relative to ApoE3 in middle-aged mice that underwent repeated mild electromagnetic controlled impact injuries for a month. They found that ApoE4 was not a risk factor for increased glial levels but that it was for increased levels of a tau marker^31^.

Consequently, the research investigating whether *APOE4* is a risk allele compared to *APOE3* after TBI is not all in agreement. Importantly, none of the existing studies have utilized the clinically relevant repeated mild lateral fluid percussion (LFP) model and compared the human forms of *APOE3* and *APOE4* in young adult mice. We specifically chose these transgenic mice in order to compare the effect that the human form of *APOE3* and *APOE4* have after injury. And while closed head models provide important information for the extremes of either very mild sub-concussive head injuries or severe head injuries with skull fracture components, using the LFP model allows us to deliver a replicable precise mild force directly to the dura. In this way, we are able to study true mild concussive hits, with a loss of consciousness in a repeated fashion; however, without the confounding factor of possible skull fractures seen in many closed head injuries. Given that certain young adult sub-populations, such as athletes and military personnel, are at an increased risk for sustaining these types of rmTBI, this investigation will yield relevant findings to real world risk factors. Furthermore, the mechanism by which *APOE4* may increase the risk for poor outcomes after TBI is not known. Recent studies have begun to investigate the role that the *APOE4* genetic polymorphism has on beneficial neurotrophic factors such as brain-derived neurotrophic factor (BDNF). It has been found that human *APOE4* carriers with Alzheimer’s disease (AD) have lower levels of BDNF in their serum^32^ than *APOE3* carriers, and that *APOE4* carriers with mild cognitively impairment have dampened BDNF increase after exercise compared to *APOE3* carriers^33^. This suggests that the *APOE4* SNP may be affecting cognition through interactions with BDNF; however, no study on the role of BDNF in outcomes after TBI has been performed for the *APOE* alleles.

In this investigation, we studied the effect of the *APOE4* genetic polymorphism on cellular, biochemical, and behavioral changes after repeated mild LFP brain injury in mice. We show that injured *APOE4* mice have increased injury area, inflammation, cell death, neurodegeneration, and activated microglia at 1 DPI in the cortex and/or hippocampus relative to injured *APOE3* mice. Injured *APOE4* mice also have increased levels of p-tau at 21 DPI compared to injured *APOE3* mice. When investigating the relative levels of BDNF after injury in these mice, we found that injured *APOE4* mice have less total BDNF than injured *APOE3* mice at 1 and 21 DPI. Finally, we have found that when injured *APOE4* mice are treated with Bryostatin-1, a PKCε activator which has been shown to increase BDNF levels^34–36^, we can rescue the elevated levels of inflammation, neurodegeneration, and activated microglia back to the levels observed in *APOE3* injured mice at 1 DPI. Bryostatin-1 is also able to rescue spatial learning at 2 DPI and spatial memory and balance at 7 DPI. This report has important implications for *APOE4* carriers, and to our knowledge, is the first report showing that this genotypical susceptibility to rmTBI can be rescued by treatment with Bryostatin-1.

## Materials and methods

### Animals

Adult male and female mice aged 10–12 weeks were used in all studies. These targeted replacement mice were developed by Patrick Sullivan and Nobuya Maeda at the University of North Carolina by targeting the murine *Apoe* gene for replacement with the human *APOE4*/*APOE3* allele^37^. Resultant chimeras were backcrossed to C57BL/6, and the colony was maintained through mating of homozygotes. Mice were housed in a 12 h light/dark cycle with food and water available ad libitum. All procedures described were performed in accordance to the NIH guidelines and were approved by the Rutgers University Institutional Animal Care and Use Committee (IACUC). A power analysis was used to determine the appropriate sample size for experiments to reach 80% power; for histology the group size n = 5–8, for biochemistry the group size n = 3–4, and for behavioral tasks n = 8–12 were used to reliability detect changes of the magnitude we are examining (α = 0.05) based on the difference seen between experimental groups in our previous publication^38,39^.

### Lateral fluid percussion injury

Lateral fluid percussion injury uses a rapid fluid pulse to cause injury to the brain by the displacement of neural tissue. This process has previously been described in detail^40^ but has been modified to create repeated mild injury^39^. Briefly, mice were anesthetized using 4–5% isoflurane in 100% O_2_ and maintained on 2% isoflurane throughout the procedure. They were placed in a stereotaxic frame, and a trephine-guide 3 mm plastic disc was attached with Loctite glue (444 Tak Pak, Henkel Corporation, Rocky Hill, CT). It was placed on the skull, halfway between lambda and bregma, laterally on the right hemisphere. A trephine (3 mm outer diameter) was used to perform a craniectomy. A rigid Luer-loc needle hub (3 mm inside diameter) was secured onto the skull over the opening that was made using cyanoacrylate adhesive and dental acrylic (Henry Schein, Dublin, OH). After a 60 min recovery period, the animals were re-anesthetized and connected to the fluid percussion injury device (Custom Design and Fabrication, Virginia Commonwealth University) through the Luer-loc hub. Once the animals regained normal breathing, before sensitivity to stimulation, a ~ 0.8 ATM pulse (15 ms) was generated through the LFP device to strike the intact dura of the brain. Upon return of righting reflex (< 4 min for mild injury) the hub was filled with saline and capped. 48 h from the initial injury, a second injury was given. This occurred again at 96 h from the initial injury. This experimental timeline was chosen based on previous studies which have sought to mimic human repeated mild TBIs in a mouse model that controls for the rodent life span^29,41–43^. While each individual hit was mild, the cumulative damage resulted in significant injury, as is seen in humans who sustain multiple concussions within a susceptibility period^44^. After the 3rd injury, the hub and dental acrylic were removed, and the scalp incision was closed with 3 M Vetbond (Fisher Scientific, Waltham, MA). The animals were individually housed after the injury and returned to normal housing conditions. In order to determine humane endpoints, the mice were monitored twice daily. If signs of pain were detected, the vivarium veterinary staff were contacted, and appropriate analgesics were used immediately. Signs of pain and distress included animals that were no longer able to move to get food or water or showed signs of pain (ex. hunched posture, inappetence, lethargy, decreased body condition). In order to prevent harm and suffering, we gave a surgical pre-emptive analgesia in the form of an injection of buprenorphrine (0.1 mg/kg SC). During the surgery, the mice were anesthetized with isoflurane while they were in the stereotaxic apparatus. Adequate anesthetic depth was checked for by a no response to toe pinch before surgery commenced. During the surgery procedure, the anesthetic bupivicaine (0.025%) was applied topically to the skull. In addition, the respiratory rate was monitored throughout the entire surgical procedure, and the eyes were protected with lubricant. If necessary, post-op pain medication of Carprofen would be given at 5 mg/kg, SC, once a day and continued if signs of pain were observed (not found to be necessary for any mice in this study). With this repeated, mild level of injury, about 5–10% of animals died after the 3^rd^ injury in the chronic post-traumatic period. In this study, we had a mortality rate of 5.03% with 15/298 deaths. The expectation of this mortality was approved by our institutional IACUC. This is a normal and anticipated feature of the LFP TBI model because it mimics human TBI. Due to the fact that the surgery itself can cause slight inflammation and in order to isolate the effects of the rmTBI, mice that underwent the surgical procedure but not the injury were used as sham controls. The sham mice also underwent the same anesthesia process as the injured mice at the 48 and 96 h timepoints. Assignment of the mice to the LFP or sham group was randomized.

### MRI imaging

Magnetic resonance imaging (MRI) was done on a first cohort of mice in order to assess the volume of edema as determined by increased relative intensity (ROI). Cohorts are described in the Supplemental Materials. (Supplemental Fig. 5) As previously described, the scans were done utilizing a fast spin echo sequence with a mouse brain coil. Scans were done in the axial position at 1 and 21 days after the final injury^39^. Edema was determined through an auto-thresholding to analyze higher intensity areas relative to regular brain tissue. All brains were reviewed with same intensity search and normalized using the Image Scale Factor in the VivoQuant Analysis Software. The region of interest was determined by analyzing areas of increased intensity within specific coordinates in the damaged location of the brain and analyzed blinded to condition. Scans were done at the Rutgers University Molecular Imaging Center with the center’s M2 Compact High-Performance MRI (1 T).

### Mesoscale discovery multiplex analysis

The cortex on the side ipsilateral to the injury site were collected from mice at 1 dpi and flash frozen as previously described from a second cohort of mice^39^. Eight mice per group and condition were analyzed at each timepoint. Tissue lysates were prepared using T-PER with protease inhibitors and EDTA (Pierce, Rockford, IL). Samples were homogenized for 30 s and then centrifuged for 10 min. The protein content of the supernatant was determined using the bicinchoninic acid (BCA) Protein Assay Reagent Kit (Pierce, Rockford, IL). Tissue lysates were analyzed using the Mesoscale Discovery V-PLEX Plus Proinflammatory Panel-1 Mouse Kit (K15048G-1; Mesoscale Discovery, Gaithersburg, MD) according to manufacturer’s instructions. Briefly, the tissue lysates were thawed at room temperature and diluted 1:2 in sample diluents. The samples were added to the plate and allowed to incubate for 2 h at room temperatures at 700 rpm. Plates were washed and allowed to incubate for 1 h with detection antibodies. After washing, the plates were analyzed using the MESO QuickPlex SQ 120 AI0AA-0; (Mesoscale Discovery).

### Immunohistochemistry

To collect tissue for immunohistochemistry, after MRI scans, mice from the first cohort were perfused with 0.9% saline, followed by 4% paraformaldehyde at 1 or 21 days after the final injury as described previously^39^. After perfusion, the brains were cryoprotected with 30% sucrose for at least 3 days. Sectioning was done in 20 µm thick slices in a 1:10 series throughout the length of the hippocampus, incorporating the area around the site of injury in the cortex. To measure apoptotic cell death, sections were pretreated with 0.01 M Citrate buffer at 90° C. Anti-cleaved caspase-3 (1:1000, 9661, Cell Signaling, Danvers, ME) was then applied overnight, followed by Alexa Fluor goat anti-rabbit 594 (1:1000, Invitrogen, Waltham, MA). To measure neuronal degeneration, sections were first treated with 1% NaOH and 0.06% KMnO_4_, then 0.0005% Fluoro-Jade C (AG325, Millipore, Burlington MA)/0.0001% DAPI (D9564, Sigma, St Louis, MO) was applied for 20 min. To measure microglial activation, IBA1 antibody was applied overnight (1:10,000, 019–19741, Wako Labs, Richmond, VA), followed by Alexa Flour goat anti-rabbit 488 (1:1000, Invitrogen, Waltham, MA). To measure levels of phosphorylated tau, AT8 antibody was applied overnight (1:500, MN1020, Pierce Antibodies, Waltham, MA), followed by Alexa Fluor goat anti-mouse 488 (1:1000, Invitrogen, Waltham, MA). All slides were incubated in 4′,6-diamidino-2-phenylindole (DAPI) (1:1000 DAPI in PBS, Sigma, St Louis, MO). Slides were mounted in Fluoromount-G (Southern Biotech, Birmingham, AL), except for the Flouro-Jade C slides which were mounted in DPX Mountant (44581, Sigma, St Louis, MO). Visualization of the fluorescent stains was done using a Leica microscope (Model DMIRB, Leica Microsystems, Buffalo Grove, IL). Five to eight animals per time point and treatment were analyzed. Sectioning of tissue was done using a Cryostat (Leica) and collected coronally in 1:10 series throughout the length of the hippocampus. For each biological replicate, the collected sections of brain were counted and the average number of cells per section was calculated. Positive cells were counted in the hemisphere ipsilateral to the injury. In the cortex, for each section, six fields of 40X view (starting at the dorsal midline and moving laterally for three fields of vision, and then the three fields of vision just ventral to the first three) were counted. In the hippocampus, the dentate gyrus as well as the CA1-CA3 were used for quantification of cells. (Supplemental Fig. 1). Analysis was performed blind to experimental group and genotype.

### Vestibular rotarod test

In order to study the vestibular motor abilities of the mice after LFP, the rotarod test was conducted as part of a behavioral battery on a third cohort of mice as previously described. ^38^ The rotarod test utilized a 36-mm outer diameter, rotating rod whose velocity increased from 4 to 40 rpm over a maximum 180 s interval. Balance and motor function were measured using the latency to fall. Each trial ended when the animal fell off the rotarod. Eight to ten mice per genotype and condition were used. Acclimation and baseline analysis were done one day prior to the first injury, using three trials separated by a one-hour inter-trial rest phase. At 1, 7, and 21 days after the last injury, each mouse underwent three trials separated by a one-hour inter-trial rest phase. The same mice were used for each time point and analyzed blinded to condition. The average latency to fall was compared between injured and sham groups.

### Balance beam test

In order to study fine motor function, the balance beam test was conducted as part of the behavioral battery on the same cohort (#3) of mice as previously described^39^. The beam apparatus consists of a one meter long flat beam with a width of 20 mm, raised 30 cm above the table surface. A black box was placed at one end of the beam as the finish point. The mice were pretested on the beam apparatus for 4 days before the test day for training and baseline measurements. On test day, the mice were observed crossing the beam while the number of paw faults, falls, and relative time to cross were recorded manually. Mice were tested at 7 and 21 DPI, and the same mice were used for each time point. Eight to ten mice per genotype and condition were used. Values were imputed into a predetermined scale to evaluate outcomes with weighted values for the different traits analyzed in order to account for the severity of injury indicated by each. A score of 1 was standard for all mice, the number of falls was added after being multiplied by 2, the number of foot faults were added, and if the mouse crossed the beam in under 5 s, a score of 1 was removed from the final score. Analysis was done blinded to condition.

### Morris water maze test

In order to study spatial memory, the Morris water maze test was done as part of the behavioral battery on the same cohort (#3) of mice as previously described^39^. Mice were acclimated to the paradigm and tested for baseline response using a visible platform test one day prior to the start of the injury paradigm. The animals were placed in a circular pool (1 m diameter) filled with opaque water containing non-toxic white paint and a clear escape platform marked by a visible rod. To assess learning, the mice were tested using a hidden platform fixed in the northeast quadrant starting one day after the last injury. Testing was conducted with four trials a day for six days in a row. On the seventh day, a probe test was completed to test memory, where the hidden platform was removed and the time spent exploring the northeast quadrant was recorded. Black and white distal extra-maze cues were positioned on the walls of the room and geometric shaped proximal extra-maze cues were positioned above the walls of the maze. The mice were placed in pseudo-randomly varied quadrants throughout testing, and the time to locate the platform was recorded. Trials were run until the mouse found the platform or was placed there after the maximum trial time of 60 s. At the conclusion of the trial, the mouse was allowed to remain on the hidden platform for 15 s to consolidate learning, followed by removal from the pool and placement onto a heating pad for 10 min. Eight to ten mice per group and condition were used. Data was analyzed blinded to condition. Data was recorded using a video-tracking system (EthoVision XT; Noldus Information Technology, Leesburg, VA).

### Western blot analysis

The cortex and hippocampus on the side ipsilateral to the injury site were collected from the second cohort of mice at 1 and 21 dpi and flash frozen as previously described^39^. For this assay, four mice per group and condition were analyzed at each timepoint. Tissue lysates were prepared using T-PER with protease inhibitors and EDTA (Pierce, Rockford, IL). Samples were homogenized for 30 s and then centrifuged for 10 min. The protein content of the supernatant was determined using the bicinchoninic acid (BCA) Protein Assay Reagent Kit (Pierce, Rockford, IL). Equal amounts of protein were loaded onto Bis Tris Gels. (Invitrogen, Grand Island, NY). The proteins were transferred onto polyvinylidene difluoride (PVDF)-filter Immobilon-P transfer membranes (Millipore, Billerica, MA). Following blocking in 5% BSA + 5% normal donkey serum overnight at 4 °C, the primary antibody was applied overnight at 4 °C. 40 µg of protein was run on a 12% Bis Tris gel and probed for pro and mature BDNF (1:500 BDNF Icosagen, San Francisco, CA). Glyceraldehyde 3-phosphate dehydrogenase (GAPDH) antibody (1:1000, Biodesign, Saco, ME) was used as a loading control. Secondary anti-mouse or anti-rabbit horseradish peroxidase (HRP)-conjugated IgG antibodies were used (1:5000, GE Healthcare, South Plainfield, NJ). GAPDH protein was visualized by chemiluminescence using the Enhanced Chemiluminescence (ECL) detection kit, (Perkin Elmer, Waltham, MA) and all others were visualized using the SuperSignal West Femto Maximum Sensitivity Substance (ThermoFisher Scientific, Waltham MA). Levels of the immunopositive bands were quantified densitometrically using Quantity One version 4.2.1 software on a GelDoc 2000 (Bio-Rad, Hercules, CA). All data is normalized to the sample’s own GAPDH and expressed as a fold change relative to the average of the genotype matched sham controls.

### Bryostatin-1 injection

Bryostatin-1 was purchased as a powder from Enzo Life Sciences (Farmingdale, NY) and reconstituted in 100% ethanol (EtOH); 1 μg of drug was dissolved in 20 μL of EtOH to make the Bryostatin-1 stock solution. The stock solution was further diluted 1:20 in PBS to make the final solution. A fourth and fifth cohort of mice was used for immunohistochemistry and Western blot at the 1 DPI timepoint. In these groups, 5 min after the final injury, mice were given a dose of 20 μg/kg intraperitoneal (i.p.) injection of the Bryostatin-1 solution or control PBS. 24 h after the first injection, mice were given a second dose of 20 μg/kg i.p. injection of either Bryostatin-1 or PBS. 3 h after the second injection, mice underwent the perfusion or tissue collection protocol. The behavioral cohort (#3) were given five doses of 20 μg/kg every 2 days beginning 5 min after the last injury. For the 21 DPI timepoint, a sixth dose of 20 μg/kg was given at 20 DPI. Dosage was done comparable to previously published literature in mice^35,45,46^.

### Statistical analysis

StatPlus software was used for all data analysis. Groups were compared using Student’s two-tailed t-test or one-way ANOVA followed by Fisher’s PLSD post-hoc analysis. p < 0.05 is considered statistically significant.

## Results

### Following rmTBI, APOE4 injured mice have more edema relative to APOE3 injured mice at 21 DPI

**Figure 1:**
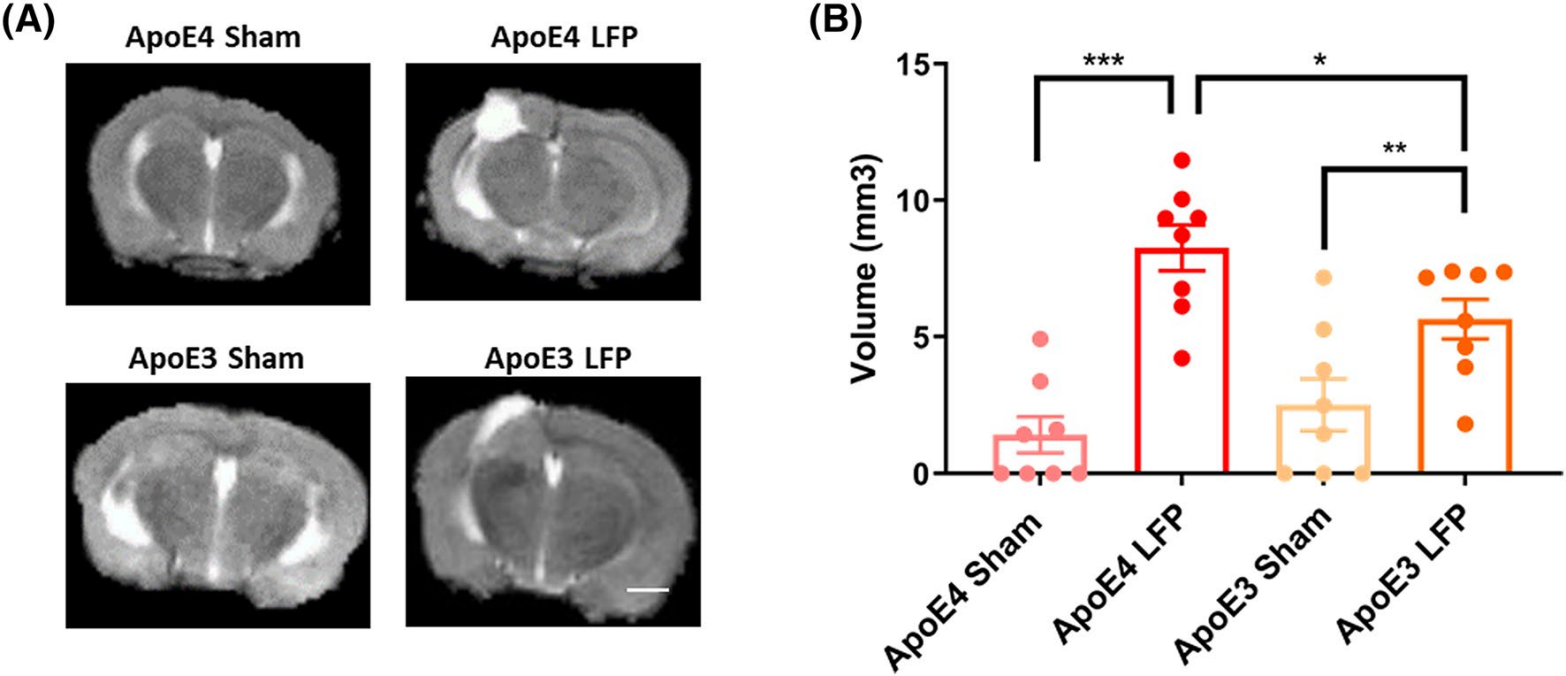
*APOE4* injured mice have greater volume of edema than *APOE3* injured mice. **(A)** Representative MRI images of mice subjected to LFP and sham at 21 days after the final injury. **(B)** Quantitation of volume of edema in different genotypes, 21 DPI as determined by assessment of T2 hyperintensity ROI. p * < 0.05, ** < 0.01, *** < 0.001, ANOVA Fisher’s PLSD post-hoc test relative to indicated groups, n = 8. Scale bar = 3 mm.

In this study, we compared the differences in outcomes between the injured and sham mice of the same genotype, as well as the differences between the two genotypes, *APOE3* and *APOE4*, when given three injuries over 5 days. Each injury was a mild concussive hit, which when given in succession resulted in significant damage, as is seen in humans who have repeated concussions within a susceptibility period^44^. In preliminary studies, we did not see any differences between the sexes, so the data from male and female mice were pooled. To investigate the role that genotype plays on edema, which can be indicative of inflammation, after repeated mild lateral fluid percussion, we scanned the mouse brains at 1, 7, and 21 DPI with a 1 T MRI and used T2 fast spin echo sequence imaging scans to conduct the analysis. By 21 DPI, we found that edema (the volume of T2 hyper-intensity at the craniotomy site) in the sham mice returned to baseline levels seen prior to the surgical procedure. Due to this, we chose the 21 DPI time point to investigate the effect of the *APOE* SNP on edema at the site of injury. We found that there was a significant difference in the volume of edema in sham relative to the injured mice. (Fig. 1A) Of note, we saw that *APOE4* injured mice had a significantly greater volume of edema relative to *APOE3* injured mice. (Fig. 1B) These results indicate that there may be differences in the recovery course of these two genotypes after injury. However, the cellular processes and molecular mechanism underlying these differences are not known.

### Inflammation is increased in APOE4 injured mice compared to APOE3 injured mice in the cortex in 1 DPI

**Figure 2:**
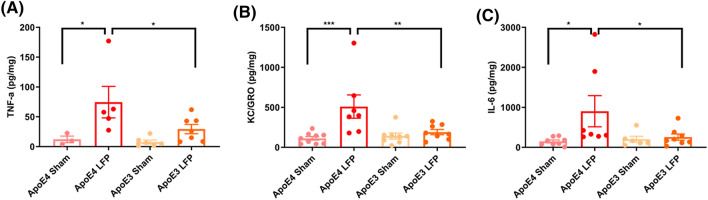
Repeated mild LFP injury causes an increase in TNF-α, CXCL1, and Il-6 in *APOE4* mice compared to injured *APOE3* mice at 1 DPI in the cortex. **(A)** Quantitation of the levels of TNF-α. **(B)** Quantitation of the levels of CXCL1. **(C)** Quantitation of the levels of Il-6. * p < 0.05, ** p < 0.01, *** p < 0.001 ANOVA Fisher’s PLSD post-hoc test relative to indicated groups, n = 5–8.

In order to better understand the cellular processes occurring after injury in these mice, we utilized multiplex enzyme-linked immunosorbent assay (ELISA) to explore changes in markers of inflammation. Previous studies have shown that various chemokines and cytokines are increased after injury. Among these are TNF-α, CXCL1 (KC/GRO), and Il-6^47,48^. Our pilot studies indicated more significant markers of inflammation in the cortex relative to the hippocampus, so we focused our analysis on the cortical samples for the ELISA. We found that after injury *APOE4* mice had significantly higher levels of TNF-α, CXCL1, and Il-6 relative to their sham controls at 1 DPI in the ipsilateral cortex. Of importance, *APOE4* injured mice also had significantly higher levels of TNF-α, CXCL1, and Il-6 in comparison to *APOE3* injured mice at 1 DPI in the ipsilateral cortex. (Fig. 2A-C) These data suggest that shortly after injury, there is a differential inflammatory response between the genotypes, with injured *APOE4* mice having increased expression of inflammatory cytokines compared to injured *APOE3* mice.

### Following rmTBI, APOE4 mice have more activated Iba1 + cells relative to APOE3 injured mice in the cortex and hippocampus at 1 DPI

**Figure 3:**
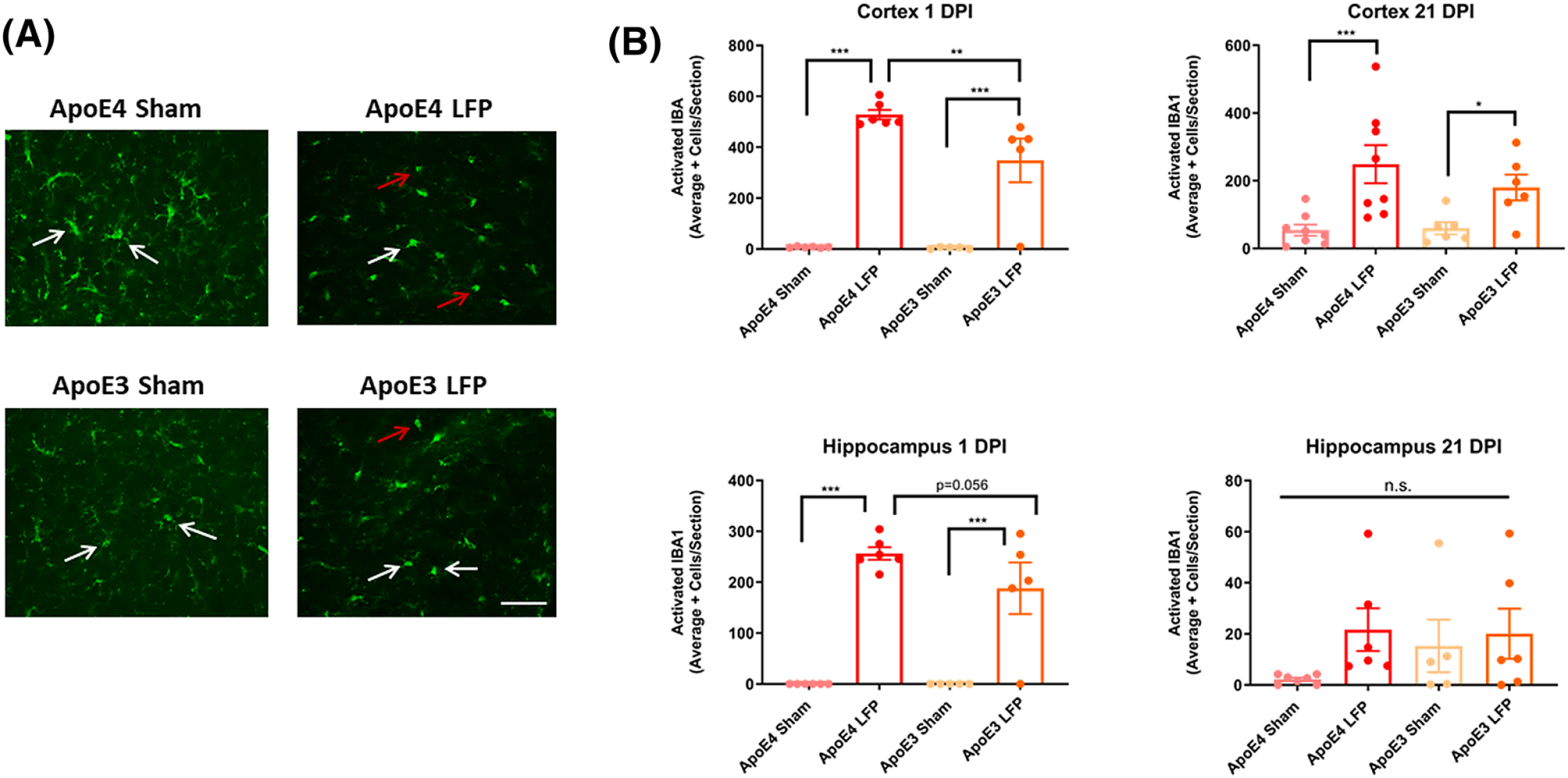
Repeated mild LFP injury causes an increase in ionized calcium binding adaptor molecule 1 (IBA1) positive cells with an activated morphology in the brains of injured *APOE4* mice compared to injured *APOE3* mice at 1 DPI in the cortex but not at 21 DPI. **(A)** Representative images of cortical sections at 1 DPI stained with IBA1, resting microglia (representative cells indicated by white arrows) and activated microglia (representative cells indicated by red arrows). Scale bars = 100 µm. **(B)** Quantitation of the average number of IBA1 + positive cells, broken down into activated and resting categories by morphology, per cortex and hippocampus ± SEM. * p < 0.05, ** p < 0.01, *** p < 0.001 ANOVA Fisher’s PLSD post-hoc test relative to indicated groups, n = 5–8.

To gain a better understanding of the cellular changes occurring, we utilized immunohistochemical staining at the 1 and 21 DPI time points to assess acute as well as longer-term outcomes after injury. We analyzed the ipsilateral cortex and hippocampus, since our lateral fluid percussion injury paradigm includes focal and distal aspects. Given that the lateral fluid percussion model of injury has diffuse components, which can have effects on the contralateral cortex and hippocampus, we used sham mice for our controls, which are mice that have had craniectomy surgery but received no injury. It has been shown that microglia activation is important part of the secondary injury process and can contribute to long-term neurological dysfunction^49^. We analyzed activated microglia to examine the effect of repeated mild traumatic brain injury on the neuroimmune system. IBA1 was used as a general marker for microglia and morphology was used to identify activated microglia from non-activated microglia. Activated microglia were distinguished by their reactive bushy or phagocytic ameboid shape, while the non-activated microglia were identified by their ramified appearance^50^. We analyzed both the number of total and activated microglia. We saw that total microglia numbers were increased in injured mice relative to their control sham mice in the cortex at 1 DPI. We also found at the 1 DPI time point, that injured *APOE4* mice had more total microglia than injured *APOE3* mice in the cortex. However, we did not see a significant effect in the hippocampus or at the 21 DPI timepoint. (Supplemental Fig. 2) Therefore, we decided to focus our analysis on the more sensitive measure of neuroimmune activation, activated microglia. At 1 DPI, we found that injured *APOE4* mice have more activated microglia than sham controls in the ipsilateral cortex and the ipsilateral hippocampus. Importantly, at 1 DPI, we found that injured *APOE4* mice have considerably more activated microglia than the injured *APOE3* mice in the ipsilateral cortex.(Fig. 3A,B) These results are indicative of an earlier microglial activation in the *APOE4* injured mice relative to the *APOE3* injured mice. At 21 DPI both the *APOE4* and *APOE3* injured mice both had increased levels of activated microglia compared to their sham controls. However, at this time point the *APOE4* injured mice no longer had increased numbers of activated microglia relative to *APOE3* injured mice. (Fig. 3A,B). This suggests that *APOE4* injured mice show a more robust early phase immune cell activation in both focal and distal areas to repeated mild injury compared to *APOE3* injured mice. This differential microglia activation has dissipated by 21 DPI, but microglia activation still remains elevated in injured mice compared to uninjured mice.

### Following rmTBI, APOE4 mice have more activated caspase-3 relative to APOE3 mice in the cortex and hippocampus at 1 DPI

**Figure 4:**
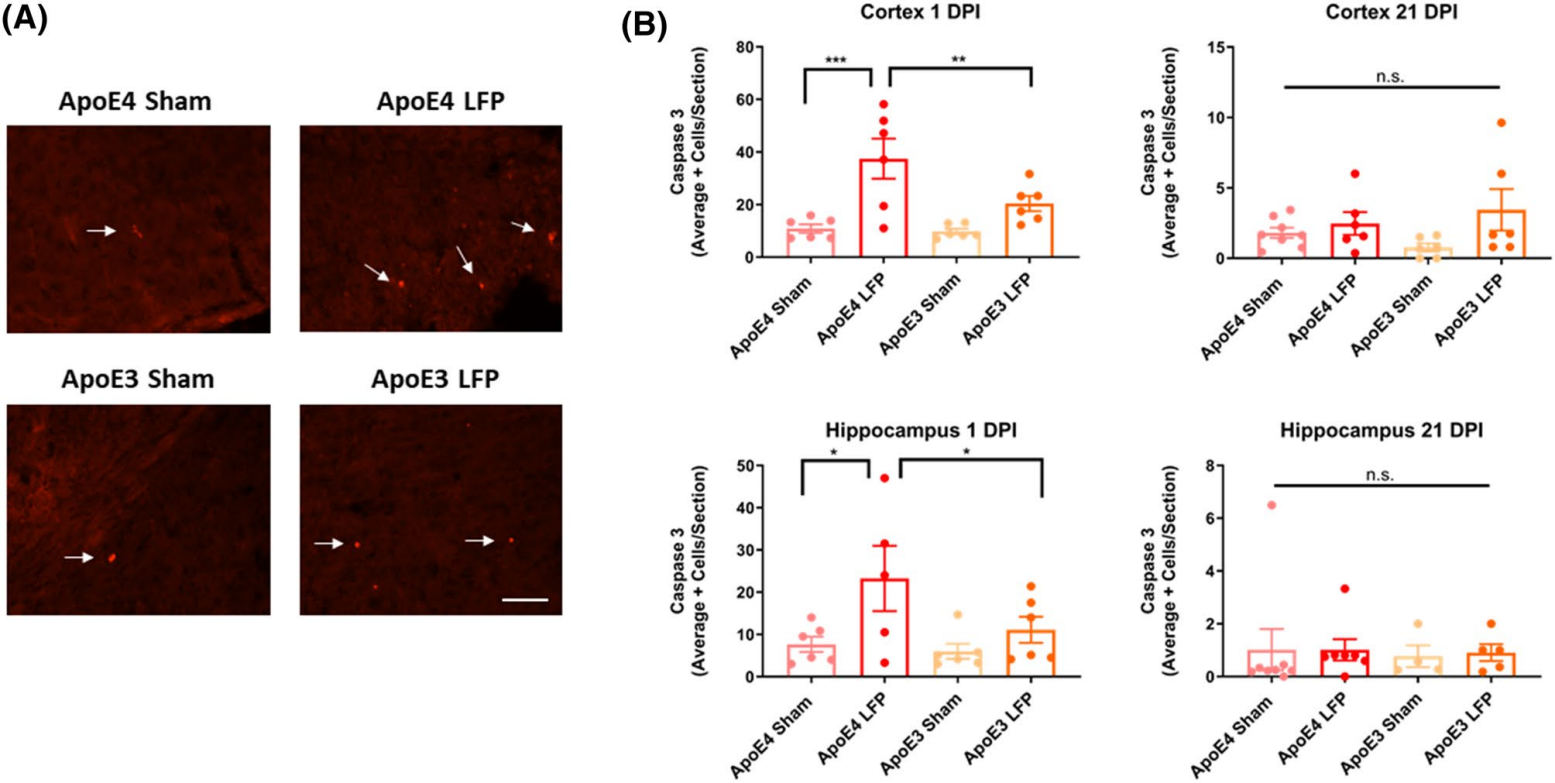
Repeated mild LFP injury causes an increase activated caspase-3 positive cells in brains of injured *APOE4* mice compared to injured *APOE3* mice at 1 DPI in the cortex and the hippocampus but not at 21 DPI. **(A)** Representative images of cortical sections at 1 DPI stained with activated caspase-3 (representative cells indicated by arrows). Scale bars = 100 µm. **(B)** Quantitation of the average number of activated caspase-3 positive cells per cortical or hippocampal section ± SEM. * p < 0.05, ** p < 0.01, *** p < 0.001 ANOVA Fisher’s PLSD post-hoc test relative to indicated groups, n = 5–8.

Activated caspase-3 was utilized in order to investigate apoptosis levels after injury, since neuronal cell death is common after injury^38,51^. At the 1 DPI time point, we saw that, compared to their sham controls, injured *APOE4* and *APOE3* mice had more activated caspase-3 positive cells, indicative of apoptosis upregulation. Of note, we saw that injured *APOE4* mice had more activated caspase-3 positive cells compared to the injured *APOE3* mice, in the ipsilateral cortex and hippocampus. (Fig. 4A,B) This indicates that *APOE4* mice have a higher occurrence of cell death than *APOE3* mice after rmTBI. Though, by 21 DPI, levels of apoptotic cell death had decreased so that injured mice did not have significantly more than sham mice. (Fig. 4A,B) These results indicate that while we see genotypic differences at 1 DPI after injury, with *APOE4* exhibiting more apoptotic cell death than *APOE3*, by 21 DPI there is some recovery and reduced apoptotic cell death in both injured groups.

### Following rmTBI, in APOE4 mice neurodegeneration is increased in relative to APOE3 mice in the cortex and hippocampus at 1 DPI

**Figure 5:**
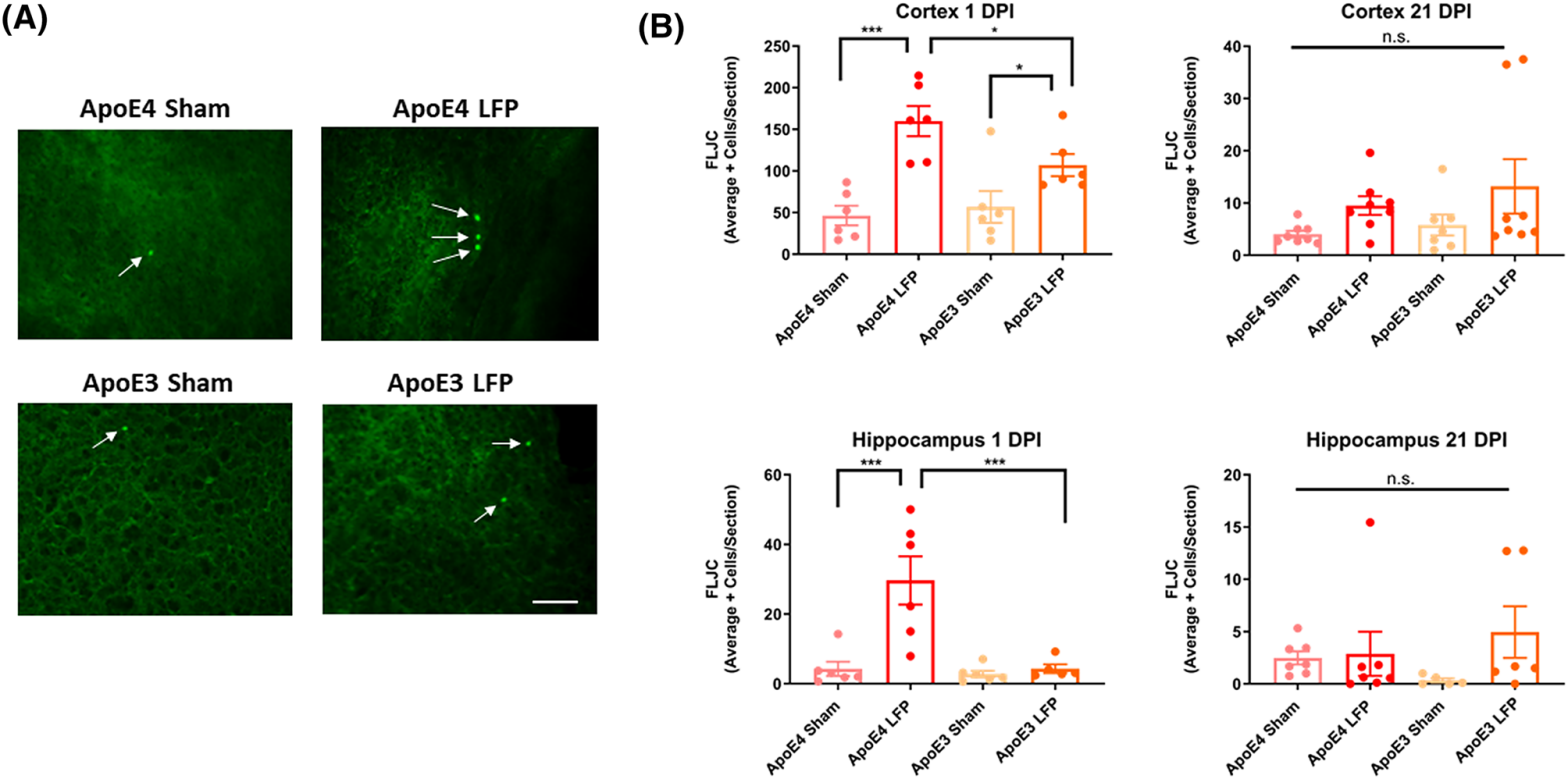
Repeated mild LFP injury causes an increase in Fluoro-Jade C (FLJC) positive cells in the brains of injured *APOE4* mice compared to injured *APOE3* mice at 1 DPI in the cortex and hippocampus but not at 21 DPI. **(A)** Representative images of cortical sections at 1 DPI stained with FLJC (representative cells indicated by arrows). Scale bars = 100 µm. **(B)** Quantitation of the average number of FLJC positive cells per cortex or hippocampus ± SEM. * p < 0.05, ** p < 0.01, *** p < 0.001 ANOVA Fisher’s PLSD post-hoc test relative to indicated groups, n = 5–8.

Another factor that is known to be upregulated after injury is neurodegeneration^38,43^. In order to investigate the levels of neurodegeneration after our injury model, we used Fluorojade C (FLJC), a well-known marker to determine levels of neurodegeneration^52^. We saw that at 1 DPI, *APOE4* injured mice had an increase in FLJC positive cells compared to sham controls in the ipsilateral cortex and hippocampus. Notably, at this time point we found that, compared to injured *APOE3* mice, injured *APOE4* mice had an increase in FLJC positive cells in the ipsilateral cortex and hippocampus (Fig. 5A,B). Though, by the 21 DPI time point, levels of neurodegeneration had returned to baseline levels so that we did not see significantly more neurodegeneration in the injured mice compared to the sham controls in either the cortex or the hippocampus (Fig. 5A,B). These results indicate that neurodegeneration is present at the early 1 DPI time point, but that the process wanes by the 21 DPI time point.

### Following rmTBI, APOE4 mice have more phosphorylated tau, relative to APOE3 injured mice at 21 DPI

**Figure 6:**
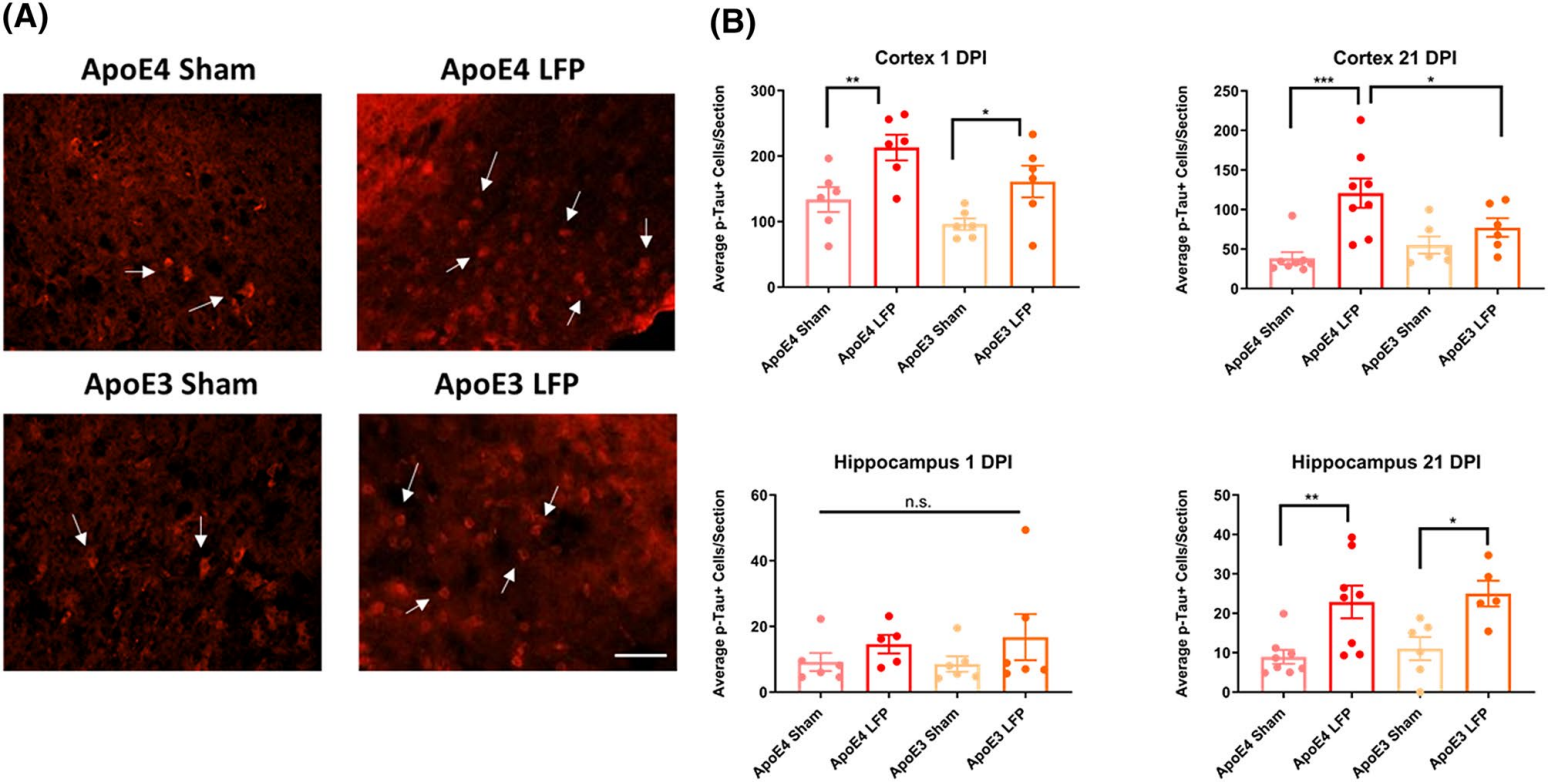
Repeated mild LFP injury causes an increase in phosphorylated tau (p-tau) in the brains of injured *APOE4* mice compared to injured *APOE3* mice at 21 DPI in the cortex. **(A)** Representative images of cortical sections at 1 DPI stained with activated AT8 (representative cells indicated by arrows). Scale bars = 100 µm. **(B)** Quantitation of the average number of AT8 positive cells per cortex or hippocampus ± SEM. * p < 0.05, ** p < 0.01, *** p < 0.001 ANOVA Fisher’s PLSD post-hoc test relative to indicated groups, n = 5–8.

Previous studies have found that after injury levels of phosphorylated tau are often increased, both in focal and distal areas of the brain^53^, which has the potential to result in long-term complications^54^. Therefore, we examined the levels of phosphorylated tau after injury, at both the 1 and 21 DPI time points, in the cortex and hippocampus. Our results show that there was more phosphorylated tau in injured *APOE4* and *APOE3* mice relative to the sham controls at 1DPI in the ipsilateral cortex. (Fig. 6A,B) At 1 DPI there was no significant difference in phosphorylated tau levels between the *APOE4* injured mice and the *APOE3* injured mice. However, by 21 DPI in the cortex the levels of phosphorylated tau in the *APOE3* injured group had returned to their sham control levels, while the *APOE4* injured levels remained elevated and significantly different from their sham controls and the *APOE3* injured group. These results suggest that injured *APOE4* mice have an exacerbated and prolonged phosphorylated tau response which may lead to long-term neurodegeneration (Fig. 6A,B).

### Injured APOE4 mice have less total BDNF than injured APOE3 mice in the cortex and hippocampus at 1 and 21 DPI

**Figure 7:**
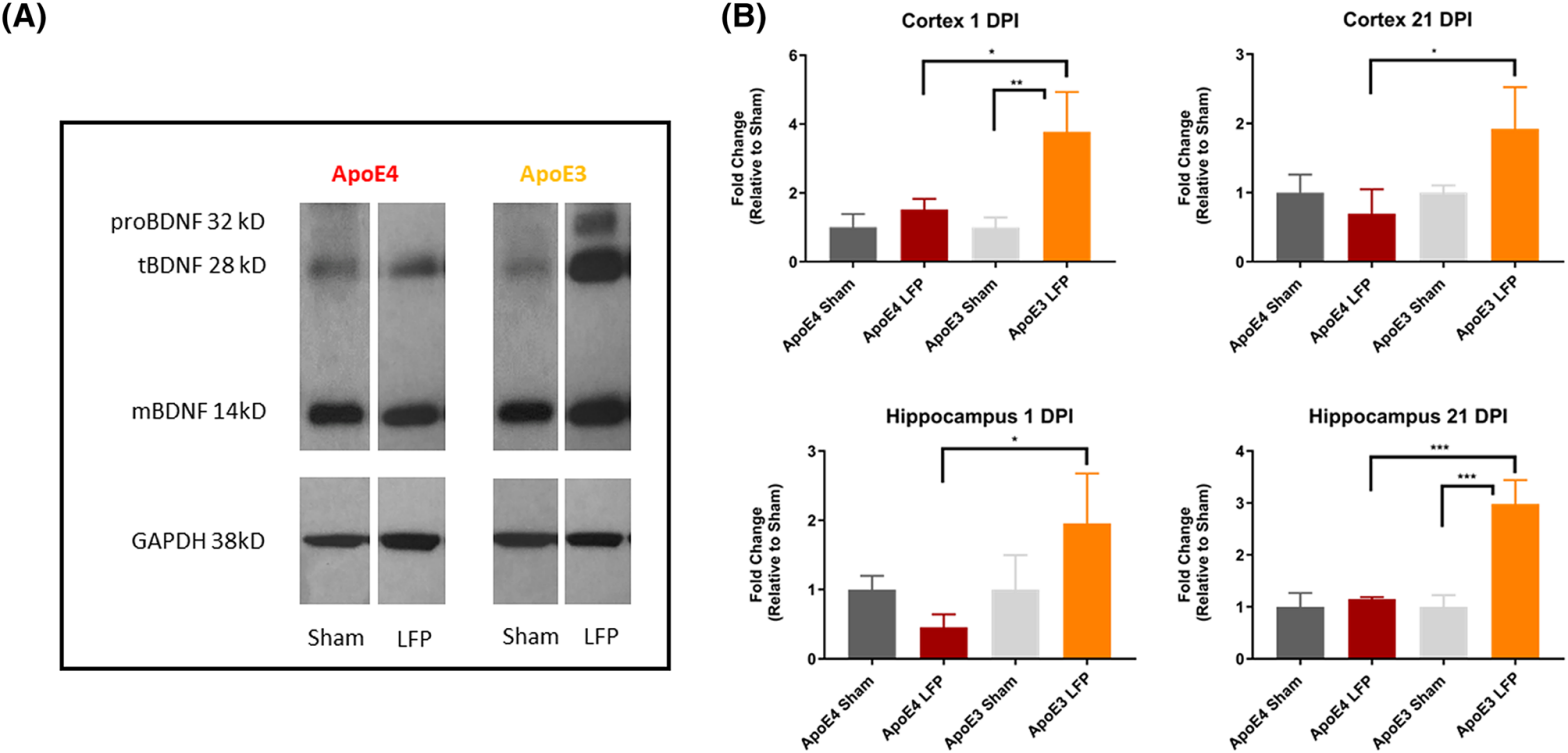
Repeated mild LFP injury causes an increase in total BDNF expression in *APOE4* mice compared to *APOE3* mice in the cortex and hippocampus at 1 and 21 DPI. **(A)** Representative Western Blot showing total BDNF expression after injury. Each lane represents one animal. Representative lanes were taken from different gels and reordered to align with the quantitative representation shown. White space between the lanes is used to delineate this point. Full length gels can be seen in Supplemental Fig. 3. **(B)** Quantitation of protein levels in the cortex and hippocampus at 1 DPI and 21. All data is first normalized to GAPDH to control for protein loading and then expressed as a fold change relative to the average ± SEM of the time matched sham controls. * p < 0.05, ** p < 0.01, *** p < 0.001 ANOVA Fisher’s PLSD post-hoc test relative to indicated groups, n = 4.

Previous studies have shown less BDNF in the serum of *APOE4* carriers with apathy^32^ and in brain tissue of *APOE4* carriers with AD^55^. It has also been shown that *APOE4* carriers have less BDNF secreted in primary hippocampal astrocyte cultures^55^. One proposed mechanism is that *APOE4* increases nuclear translocation of histone deacetylases, which has the downstream effect of reducing BDNF levels^56^. However, to our knowledge, no one has investigated levels of BDNF in the different *APOE* genetic polymorphisms after rmTBI. We found that after injury, levels of total BDNF are significantly higher in *APOE3* injured mice relative to their sham control in the cortex and hippocampus at 1 and 21 DPI using Western Blot analysis. (Fig. 7A,B, Supplemental Fig. 3) Of importance, we also saw that there was a significantly more total BDNF in *APOE3* injured mice than there was in *APOE4* injured mice, and that all forms of BDNF (pro, truncated, and mature) were elevated in these mice. As is common with BDNF Western Blot analysis, we also saw non-specific bands in the higher molecular weight regions^38^. These data support our conclusion that there is a differential neurotrophic response to injury in *APOE3* compared to *APOE4* carriers and highlight a potential pathway to target to therapeutics.

### Treatment of APOE4 injured mice with Bryostatin-1 reduces the excessive inflammation and neurodegeneration seen in the injured APOE4 mice back to the levels present in the injured APOE3 mice at 1 DPI

**Figure 8:**
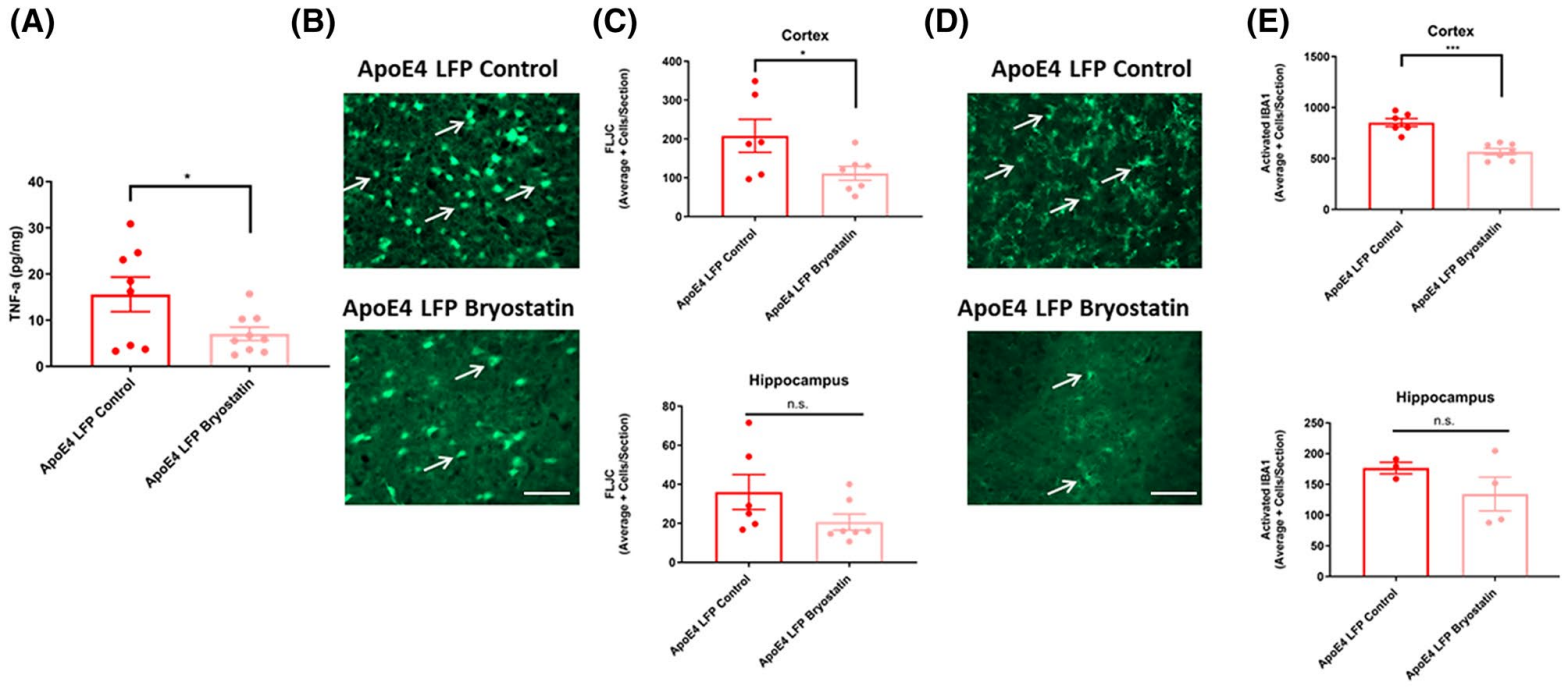
Treatment of injured *APOE4* mice with Bryostatin-1 after repeated mild LFP injury causes a significant decrease in TNF-α in the cortex, astrogliosis in the cortex and hippocampus, and activated microglia in the cortex at 1 DPI. **(A)** Quantitation of the levels of TNF-α in the cortex at 1 DPI. **(B)** Representative images of cortical sections at 1 DPI stained with FLJC. Scale bars = 100 µm. **(C)** Quantitation of the average number of FLJC positive cells per cortex and hippocampus ± SEM. **(D)** Representative images of cortical sections at 1 DPI stained with IBA1. Scale bars = 100 µm. **(E)** Quantitation of the average number of IBA1 positive cells per cortex and hippocampus ± SEM. * p < 0.05, ** p < 0.01, *** p < 0.001 Student’s t-test relative to indicated groups, n = 6–9.

Analysis of our ELISA and immunohistochemical data suggests that *APOE4* injured mice have worse recovery after rmTBI, especially in inflammation (TNF-α and activated IBA1) and neurodegeneration (FLJC) at 1 DPI than their sham controls and *APOE3* injured mice. In order to determine if we can treat *APOE4* carriers with a personalized therapy to ameliorate this risk, we used Bryostatin-1, a PKCε activator, which has previously been used to treat *APOE4* carriers with AD^35^, after stroke^57^, and after blast injury^58^. We found that when treated with two doses of 20 µg/kg Bryostatin-1, *APOE4* injured mice had significantly less TNF-α, fewer FLJC + cells, and fewer activated IBA1 + cells at 1 DPI in the cortex than *APOE4* injured mice treated with control PBS. These data suggest that Bryostatin-1 may be a potent treatment option for improving cellular outcomes in injured *APOE4* carriers (Fig. 8A–E).

### Treatment of APOE4 injured mice with Bryostatin-1 improves fine motor balance at 7 DPI, but not gross vestibular motor function, back to levels seen in APOE3 injured mice

**Figure 9:**
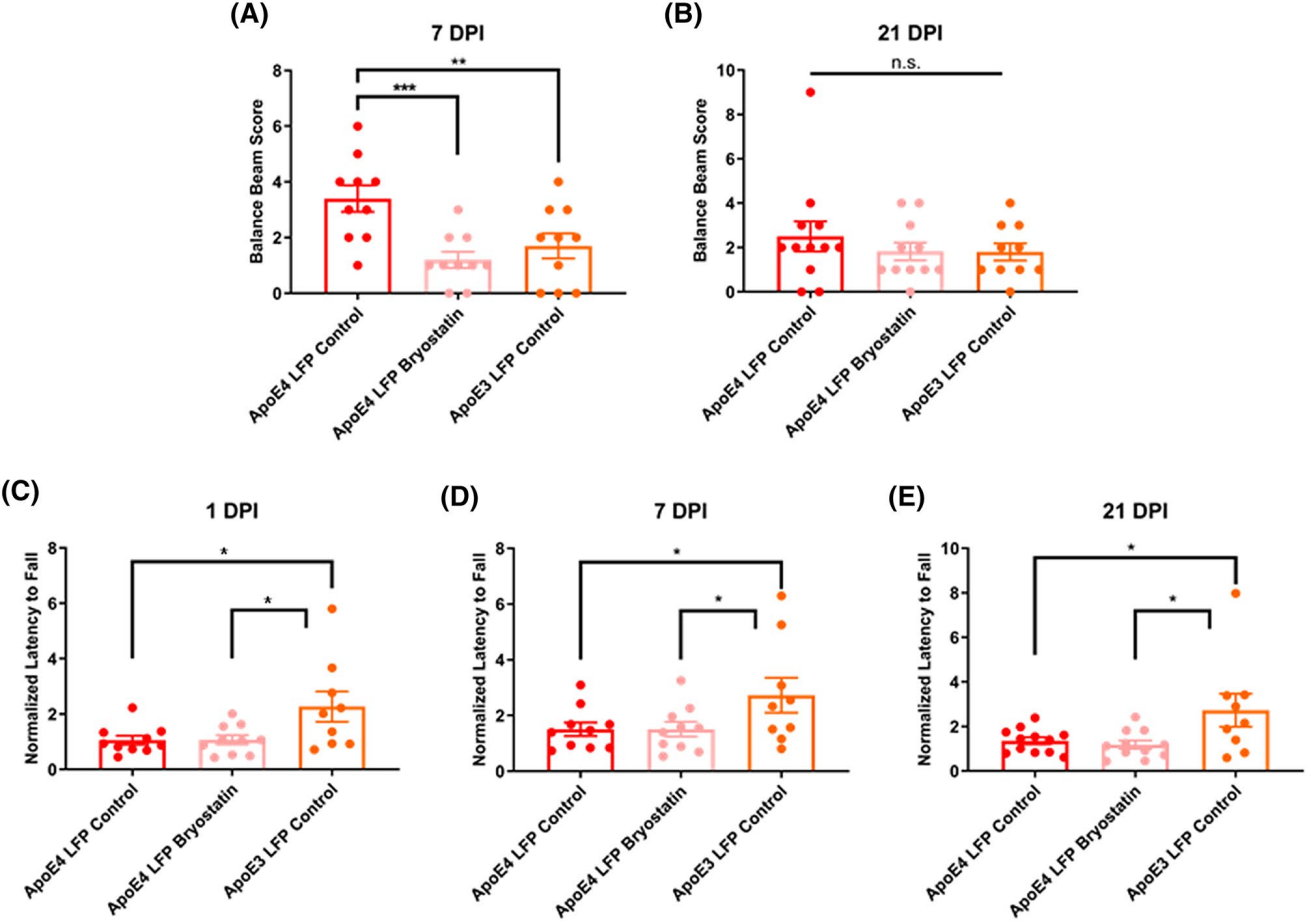
Repeated mild LFP injury causes impaired fine motor balance and vestibular motor function in injured *APOE4* mice relative to injured *APOE3* mice. At 7 DPI, treatment of injured *APOE4* mice with Bryostatin-1 improves balance but not vestibular motor function. **(A)** Quantitation of balance beam score at 7 DPI and **(B)** 21 DPI. **(C)** Quantitation of the normalized latency to fall in the Rotarod assay at 1 DPI, **(D)** 7 DPI, and **(E)** 21 DPI. * p < 0.05, ** p < 0.01, *** p < 0.001 ANOVA Fisher’s PLSD post-hoc test relative to indicated groups, n = 9–10.

It has previously been shown that following brain injury in mice, there can be deficits in motor ability^59,60^. This is particularly true with our model of lateral fluid percussion due to the damage that is done to the sensorimotor cortex at the site of injury^61^. In order to measure motor ability, we used the Balance Beam Test for subtle differences in motor skills and balance and the Rotarod Test for vestibular motor function and proprioception. For the Balance Beam Test, we found that *APOE4* vehicle control injured mice had a higher balance beam score, indicative of worse balance, than the *APOE3* vehicle control injured mice at 7 DPI. By 21 DPI balance beam scores decreased so that there were no significant differences between groups. (Fig. 9A,B) Of note, we found that *APOE4* control injured mice treated with 5 doses of 20 ug/kg of Bryostatin-1 had a significantly decreased balance beam score at 7 DPI, indicating that treatment with Bryostatin-1 was able to improve fine motor balance skills. For the Rotarod Test, we found that *APOE4* vehicle control injured mice had a shorter latency to fall relative to *APOE3* control injured mice at 1, 7, and 21 DPI, indicating impaired vestibular motor function. However, we found that *APOE4* injured mice treated with Bryostatin-1 did not have significantly different latency to fall than their *APOE4* injured control treated counterparts, suggesting that treatment with Bryostatin-1 does not rescue gross vestibular motor skills (Fig. 9C-E).

### Treatment of APOE4 injured mice with Bryostatin-1 improves learning at 2 DPI and memory at 7 DPI back to levels seen in APOE3 injured mice

**Figure 10:**
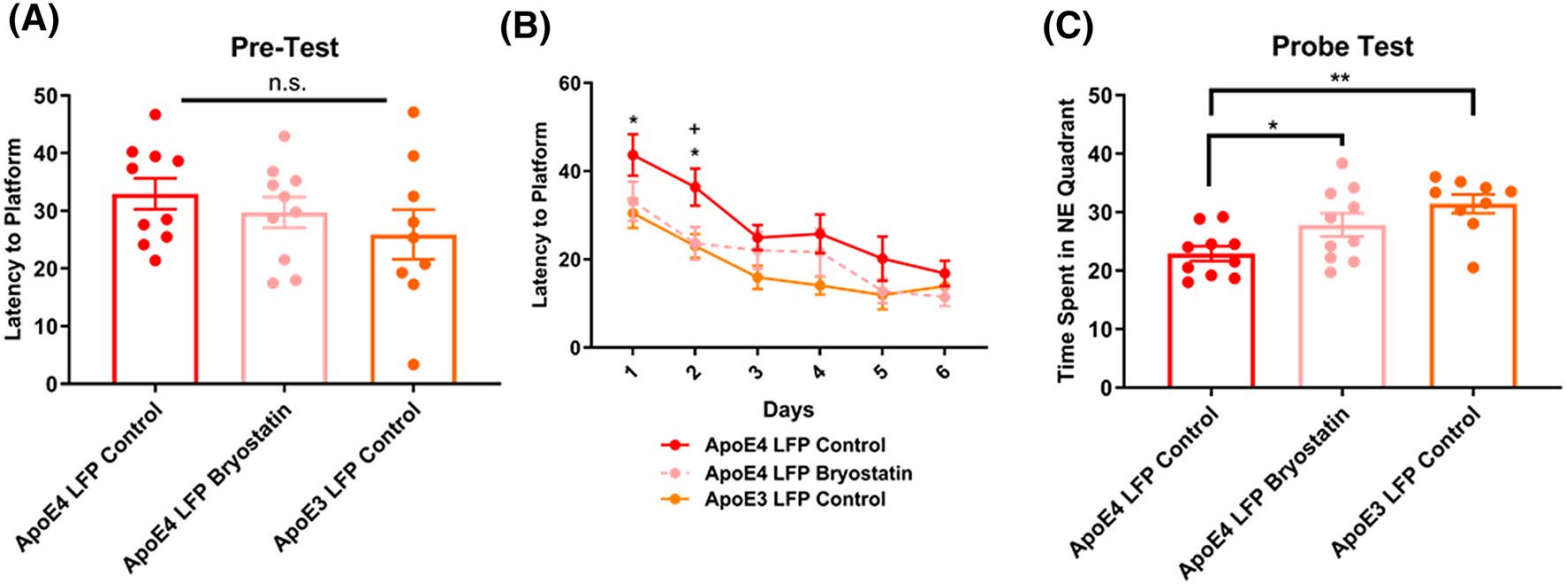
Repeated mild LFP injury causes worse learning and memory in *APOE4* injured mice relative to *APOE3* injured mice. Treatment with Bryostatin-1 in injured *APOE4* mice improves learning 1 and 2 DPI and memory at 7 DPI. **(A)** Average latency to platform in pretest phase ± SEM. **(B)** Average latency to platform ± SEM 1–6 DPI. **(C)** Average time spent in the target quadrant in the probe test ± SEM at 7 DPI. * p < 0.05, ** p < 0.01, *** p < 0.001 in *APOE4* LFP Control relative to *APOE3* LFP Control, + p < 0.05, +  + p < 0.01, +  +  + p < 0.001 in *APOE4* LFP Control relative to *APOE4* LFP Bryostatin-1. ANOVA Fisher’s PLSD post-hoc test relative to indicated groups, n = 9–10.

Finally, we examined the effect of Bryostatin-1 treatment after injury in *APOE4* mice on cognitive function using the Morris Water Maze to study spatial learning and memory. Previous studies have established that brain injury can have detrimental effects on cognition, especially in animals that have sustained injury to the hippocampus^38,59^. In our pretest before injury, we found no baseline differences between groups. (Fig. 10A) In the training stage after injury, we found that *APOE4* vehicle control injured mice had a longer latency to find the hidden platform compared to *APOE3* vehicle control injured mice at 1 DPI and 2 DPI, suggesting impairment in spatial learning. Importantly we found that when treated with five doses of 20 ug/kg of Bryostatin-1, *APOE4* injured mice found the hidden platform in significantly less time than their *APOE4* vehicle control injured counterparts at 2 DPI. (Fig. 10B) In the Probe Trial at 7 DPI, we found that *APOE4* control injured mice spent less time in the target NE quadrant than *APOE3* control injured mice, indicating impaired spatial memory. Importantly, we found that *APOE4* Bryostatin-1 injured mice spent significantly more time in the target NE quadrant than the *APOE4* control injured mice. (Fig. 10C) These data suggest that treatment with Bryostatin-1 is able to rescue learning and memory deficits in vulnerable *APOE4* injured mice following rmTBI in addition to improving immunohistochemical differences.

### Treatment of APOE4 injured mice with Bryostatin-1 does not significantly increase total BDNF levels at 1 DPI relative to control treatment

**Figure 11:**
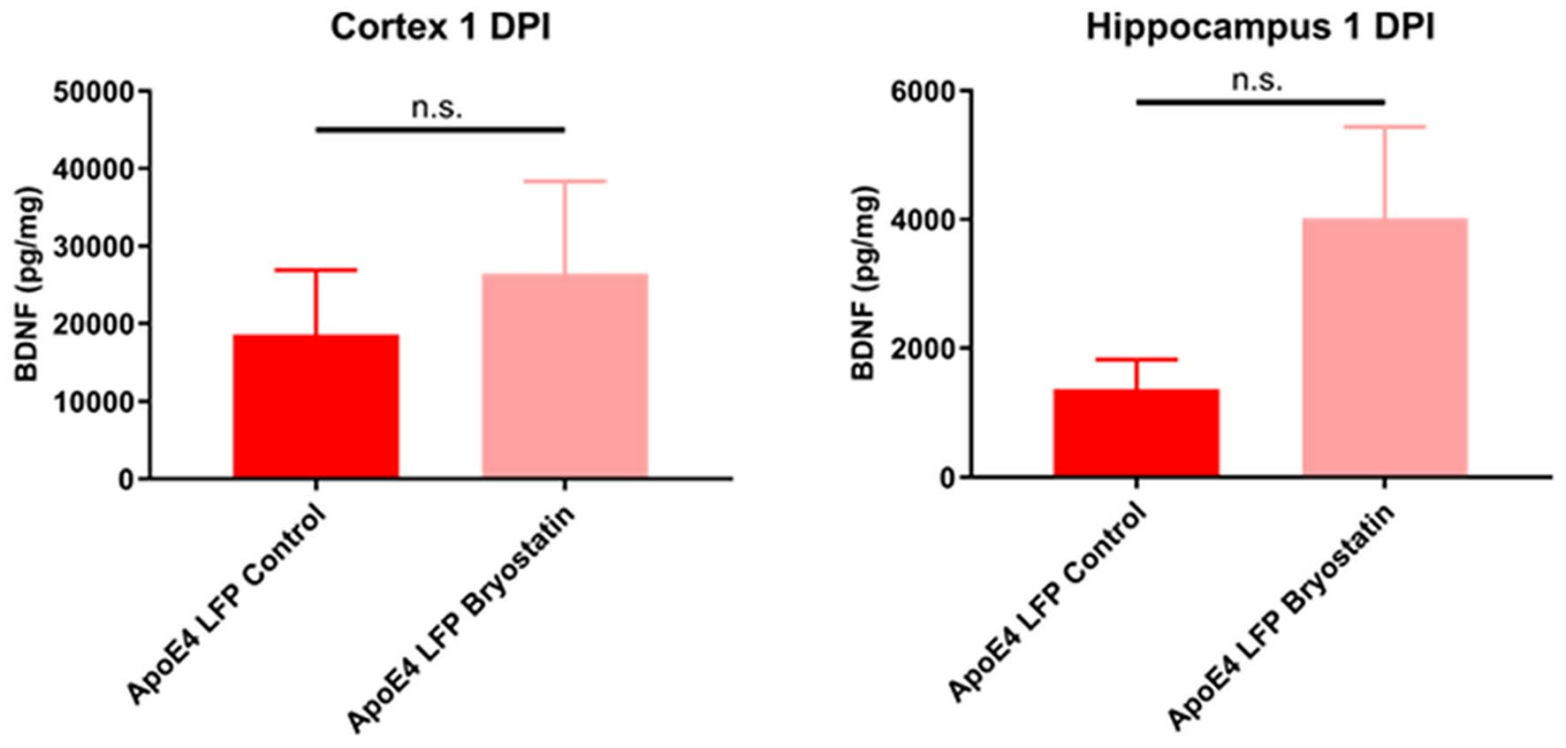
Treatment with Bryostatin-1 in injured *APOE4* shows a trend to increased BDNF levels at 1 DPI. Quantitation of BDNF levels of injured Bryostatin-1 and control mice lysates from the cortex and hippocampus at 1 DPI by ELISA. Average BDNF levels are measured in concentration units of pg/mg ± SEM. Student’s t-test relative to indicated groups, n = 8–9.

In order to determine if Bryostatin-1 was having its beneficial effects at 1 DPI through increasing BDNF levels as previously shown^35^, we decided to conduct Multiplex ELISA analysis on tissue from the cortex and hippocampus, given that this has been shown to be a more sensitive assay than Western Blot analysis. We found that at the early 1 DPI timepoint, levels of total BDNF in the Bryostatin-1 treatment group trended to increase compared to the control treatment group, although this difference was not significant. (Fig. 11). From this, we conjecture that the early beneficial actions of Bryostatin-1 after injury may be independent from BDNF.

## Discussion

In this study, we report that the *APOE4* genetic polymorphism exacerbates the injury outcome relative to the *APOE3* genetic polymorphism after rmTBI in a mouse model. We used the LFP model of injury because it is able to recreate injuries similar to those seen in the human population with both focal and diffuse components^38,40,62,63^. In order to gain an appreciation for the biological processes that are activated at different time points after injury, we investigated cellular and biochemical changes at 1 and 21 DPI in both the ipsilateral cortex and hippocampus, to examine focal and diffuse effects of injury respectively. We saw the most dramatic effects in the cortex, presumably due to the fact that this was the primary site of injury.

Previous work has shown that ApoE plays an important part in brain recovery after injury due to its support of neuronal repair mechanisms^64–66^. After injury, ApoE has been shown to play a role in the recovery of the blood brain barrier (BBB)^67^, the clearance of neurotoxic fragments^68^, and suppressing pro-inflammatory factors^69^. In fact, studies have shown that ApoE mimetics are able to ameliorate detrimental neurodegeneration seen in an AD mouse model^70^. However, there appears to be differences in how effectively the isoforms of ApoE are able to complete these tasks. Prior studies have shown that in the brain, ApoE4 protein may be less effective than ApoE3 in repairing the BBB, clearing neuronal fragments, and suppressing pro-inflammatory factors, and proteolytic cleavage of ApoE4 may actually contribute to the collection of neurotoxic fragments that can result in long-term pathology^70^. However, it is less well understood how these genetic polymorphisms play a role in acute recovery after rmTBI and if there are any chronic effects that can be seen after this injury paradigm.

We have demonstrated that injured *APOE4* mice have more cells undergoing apoptotic cell death and neurodegeneration in the acute phase of injury than the injured *APOE3*. Previous studies have shown that elevated levels of apoptosis and neurodegeneration are tied to worse outcomes after injury^52,71,72^. It has also been shown that ApoE4 results in elevated levels of apoptosis^73^ and neurodegeneration^74^ relative to ApoE3 acutely after injury. However, we have shown that in the chronic phase of injury, levels of apoptotic cell death and neurodegeneration in the injured ApoE4 mice returned back to sham baseline conditions. This is consistent with previous reports^75,76^ and indicates that other pathways may play a role at later time points in the development of chronic injury processes.

In addition to apoptosis and neurodegeneration, there is an increase in inflammation and activation of the neuroimmune system acutely after injury. By MRI analysis, we were able to show that at 21 DPI the *APOE4* injured mice have a larger volume of edema than the *APOE3* injured mice, possibly indicating increased inflammation. By Multiplex and immunohistochemistry analysis, we were able to show that highly activated factors in *APOE4* injured mice include CXCL1, Il-6, TNF-α, and microglia. CXCL1 is a cytokine that is a ligand for the CXCR2 receptor, whose activation has been implicated in increased pain after injury^77^ and blockade has been shown to reduce neuroinflammation after injury^78^. Il-6 is an important pro-inflammatory interleukin. Increased levels have been correlated with poor outcomes after injury^79^, and its blockade decreases this effect^80^. TNF-α is an important pro-inflammatory cytokine that is released after injury. Previous work has shown that TNF-α inhibits neurite outgrowth after injury^81^, genetically manipulated mice without TNF-α have improved outcomes after injury^82^, and treatment with the drug etanercept, which blocks TNF-α activity, improves outcomes after injury^83^. Importantly, TNF-α also has been implicated in activating microglia and astrocytes^84^. Microglia are the resident neuroimmune cells of the brain and play an important role in maintaining homeostasis in the brain. After injury, they are activated in order to initiate the neuroimmune response to injury^85,86^. Previous work has highlighted the role that increased activation of the immune system can play when interacting with the ApoE isoforms, with the ApoE4 isoform stimulating pro-inflammatory factors^87^ and the ApoE3 isoform promoting a reduced M1 microglia activation^88^. In particular, recent work has shown that microglia play an important role in driving *APOE* related neurodegeneration in tau-AD models^89^. We found that at 1 DPI in the ipsilateral cortex, *APOE4* injured mice have significantly higher levels of CXCL1, Il-6, TNF-α, and elevated activated microglia. This suggests that differential elevation of pro-inflammatory cytokines and activated microglia may play a role acutely after injury, initiating sequelae that may contribute to the chronic differences between the two genotypes.

One important contribution to long-term neurodegeneration is the hyper-phosphorylation of tau. After injury, tau can become hyper-phosphorylated, which allows it to bind to other tau proteins and form aggregates. These aggregates can disrupt normal functioning in the brain and lead to chronic neurodegeneration^29,90,91^. We have shown that the levels of phosphorylated tau in injured *APOE4* mice remain elevated at 21 DPI. These data highlight phosphorylated tau as a potential cause of long-term neurodegeneration in *APOE4* injured mice and differential outcomes between injured *APOE4* mice and injured *APOE3* mice. These results are consistent with previous work that has shown that *APOE4* carriers have impaired autophagy and clear tau more slowly^92^. It is known that the hyper-phosphorylation of tau increases with the combination of the ApoE4 isoform and age^93^, highlighting the role it may play in the development of the neuronal deficits seen in Alzheimer’s Disease. Importantly, we show here that rmTBI in young adults can also stimulate this relative increase in phosphorylated tau in injured *APOE4* mice compared to injured *APOE3* mice.

Based on the results presented in this study, we conclude that the *APOE4* genetic polymorphism is a risk allele for poor outcomes after rmTBI. These data are consistent with previous literature about the role of *APOE4* genetic polymorphisms in rmTBI models. Previous research has found that after repeated blast injury, ApoE4 injured mice had more p-tau regulated by synj1, an important regulatory lipid phosphatase, relative to ApoE3 injured mice^30^. Another recent study examined the effect of ApoE4 relative to ApoE3 in 12-month-old “middle-aged” mice that underwent repeated mild injuries for a month. They found that ApoE4 was not a risk factor for increased astrogliosis or total microglia levels, but it was for increased levels of MC1, a tau conformational marker^31^. One group found that ApoE was necessary for the proliferation of hippocampal neural stem cells after injury, and that the human *APOE* genotype seemed to influence this process^94^. Yet another group attempted to investigate the role that ApoE4 has on recovery after a repeated mild CCI injury and found no effect of the ApoE4 genotype on recovery in their model. However, given the parameters they used, which involved a very mild injury, it is possible that they were unable to distinguish any small differences that may be occurring. In addition, they used WT C57BL/6 mice as the control mice, which have the mouse gene sequence of ApoE4. Since these mice have the ancestral allele, which is homologous to *APOE3*, the study is not an accurate comparison of the difference between the human *APOE3* and *APOE4* alleles^29^.

Considering there seems to be a genotypic difference between the two isoforms and how they recover after rmTBI, it is necessary to investigate the pathway through which these differences may be mediated. Previous studies have postulated that the mechanism may be due to the role that ApoE plays in neural repair, with ApoE4 carriers having impaired ability to repair membranes after injury, while others have suggested that it is due to the neurotoxic fragments that are created when ApoE4 is proteolytically cleaved^64–66^. Recent studies have begun to investigate the role that the ApoE4 genetic polymorphism has on neurotrophic factors in the brain. Studies have found that AD *APOE4* carriers have lower levels of BDNF in the serum^32^, and that mildly cognitively impaired *APOE4* carriers have dampened BDNF increase after exercise compared to non-carriers^33^. Interestingly, it has been found that *APOE4* causes reduced BDNF release by increasing the nuclear translational of histone deacetylases (HDAC) 4/6. This action causes negative gene regulation, and results in less BDNF being produced in the cell and secreted by astrocytes^55,56^. It is known that BDNF signaling plays an important role in recovery after injury^38,95^. We have previously shown that treatment with overexpression of BDNF in susceptible BDNF Val66Met mice was able to improve outcomes after rmTBI^39^. This suggests that BDNF may be a potential therapeutic target for *APOE4* carriers. Our data that injured *APOE4* mice have lower levels of total BDNF than injured *APOE3* mice highlight the role that alterations in BDNF signaling may play in differential outcomes after injury seen in *APOE* mice.

The investigation of Bryostatin-1, an activator of PKCε when given in low doses over short time courses, is currently underway as a treatment option for AD^35^. Bryostatin-1 has been shown to increase BDNF levels and improve cognitive ability in mouse studies performed in an AD model, showing promise that it may be effective for improving outcomes in *APOE4* carriers after injury as well. For this reason, we chose to use Bryostatin-1 as a personalized treatment for injured *APOE4* mice. Importantly, we found that treatment with Bryostatin-1 was able to reduce the elevated levels of neurodegeneration, TNF-α, and activated microglia in injured *APOE4* mice relative to control injured *APOE4* mice. Bryostatin-1 treatment also was able to increase learning and memory as well as improve fine motor balance but was unable to improve gross vestibular motor ability. This may be due to the fact that the balance beam test is a more sensitive assay and was able to detect differences not seen in the Rotarod test. Interestingly, we did not find that treatment with Bryostatin-1 significantly increased levels of BDNF at our 1 DPI timepoint as analyzed by Multiplex ELISA. Future experiments will investigate alternative dosing levels and schedules that might result in Bryostatin-1 treatment increasing BDNF levels as expected from previous studies^35^. Our data suggest that Bryostatin-1 is able to improve outcomes after rmTBI, presumably by its activation of PKCε, and its action on positive gene regulation.

One key aspect of our study is that it was done in a clinically relevant population, and our treatment plan was informed by clinically relevant literature. We used mice at 10–12 weeks, the equivalent of young adulthood in humans, and an age where certain sub-populations such as athletes and military personnel are at increased risk for sustaining rmTBI^3,96^. We did not initiate the treatment protocol until after the final injury, as would be expected in the human rmTBI population, and gave clinically appropriate doses^35^. Therefore, this study lays the groundwork for understanding the differential outcomes seen after rmTBI in *APOE* genetic polymorphic mice and highlights the potential that personalized therapies may be able to play in ameliorating symptoms and improving outcomes.

Our study was limited by a number of factors that future studies should attempt to improve upon. We selected the 1 and 21 DPI time-points based on indications from our previous studies with single moderate TBI. However, it is known that rmTBI may have long-term effects. Future studies may benefit from including outcomes such as 6 months to 1 year. We used the LFP model in order to give replicable true mild TBI hits, however it must be noted that this is an open head model. While the LFP model offers certain scientific benefits over closed head models, it does not exactly replicate how human TBIs occur. Future studies would attempt to repeat these experiments in a closed head model as well. Moreover, we chose to use a very controlled experimental paradigm to investigate the effect of one singular genetic polymorphism on outcomes after rmTBI. This afforded us benefits in limiting confounding factors but does not take into account the complex genetic background that many people who sustain rmTBI have. Upcoming studies would be wise to investigate the role of other genetic polymorphisms in combination with *APOE4*, such SNPs that exist in the BDNF gene to determine if these genetic polymorphisms together act to compound the damage seen after rmTBI. In addition, we followed a limited Bryostatin-1 dosing regimen in only the injured ApoE4 group due to expense of the compound. In future studies, we will expand our Bryostatin-1 treatment dosing regimen and groups, taking into account that previous work has shown that levels of BDNF increase with increased Bryostatin-1 dosing and at later time points^35,36,57,58,97^. Specifically, previous studies have shown that BDNF levels peak in mice after three weekly injections of Bryostatin-1 at 25 μg/m^2^^35^, which might be a more optimal dosing protocol. However, given that we saw beneficial effects of Bryostatin-1 at our 1 DPI timepoint, but no increase in BDNF levels, we also will investigate other mechanisms through which it may be having its beneficial effects, such as on other downstream targets of PKCε or TNF-α. Future studies could consider using anti-inflammatory drugs that block the actions of TNF-α, such as etanercept^83^, or other targeted therapies, such as *APOE*-mimetic treatment, which has shown promise in treating TBI in *APOE4* animal models in previous studies^24^.

To conclude, this study has investigated the role of the *APOE4* genetic polymorphism on outcomes after rmTBI such as inflammation volume, cellular markers, protein levels, and behavior. We have explored the use of Bryostatin-1 as a personalized treatment method in these mice and have shown that it is able to alleviate some of the damage seen after rmTBI. This report highlights *APOE4* as a risk factor for poor outcomes after rmTBI and the potential usefulness of PKCε activators for treatment in this genotype.

## Supplementary information

Supplementary Figures.
