# Supplementary material for: APOE4 genetic polymorphism results in impaired recovery in a repeated mild traumatic brain injury model and treatment with Bryostatin-1 improves outcomes
Source: Sci Rep. 2020 Nov 16;10:19919. doi: 10.1038/s41598-020-76849-x (PMC7670450; doi:10.1038/s41598-020-76849-x)
Supplement: Supplementary file 1 — Supplementary Figures. [file 41598_2020_76849_MOESM1_ESM.docx]

***APOE4* genetic polymorphism results in impaired recovery in a repeated mild traumatic brain injury model and treatment with Bryostatin-1 improves outcomes**

Anna O. Giarratana ^a^, Cynthia Zheng ^a^, Sahithi Reddi ^a^, Shavonne Teng ^a^, David Berger ^a^, Derek Adler ^a^, Patrick Sullivan *^b^*, Smita Thakker-Varia ^a^ *, Janet Alder ^a^ *

*^a^ Department of Neuroscience and Cell Biology, Rutgers Robert Wood Johnson Medical School, Piscataway, New Jersey; ^b^ Department of Medicine, Duke University, Durham, North Carolina*

* Co-senior authors. Correspondence should be addressed to Janet Alder, Ph.D. Department of Neuroscience and Cell Biology, Rutgers Robert Wood Johnson Medical School, 675 Hoes Lane West, Research Building 357A, NJ 08854-5635. Tel: 732-235-5392. E-mail: [janet.alder@rutgers.edu](mailto:janet.alder@rutgers.edu)

Anna O. Giarratana, PhD

Post-Doctoral Researcher – Medical Student

Rutgers Robert Wood Johnson Medical School

675 Hoes Lane West

Piscataway, NJ 08854

Email: giarraan@rwjms.rutgers.edu

Tel: 732-235-5394

Fax: 732-235-4990

Cynthia Zheng, BA

Medical Student

Rutgers University

675 Hoes Lane West

Piscataway, NJ 08854

Email : cz204@scarletmail.rutgers.edu

Tel: 732-235-5394

Fax: 732-235-4990

Sahithi Reddi, BA

Student Research Technician

Rutgers Robert Wood Johnson Medical School

675 Hoes Lane West

Piscataway, NJ 08854

Email: sahithireddi@yahoo.com

Tel: 732-235-5394

Fax: 732-235-4990

Shavonne Teng, BS

Graduate Student

Rutgers Robert Wood Johnson Medical School

675 Hoes Lane West

Piscataway, NJ 08854

Email: shtengus2@gmail.com

Tel: 732-235-5394

Fax: 732-235-4990

David Berger, BA

Research Assistant

Rutgers Robert Wood Johnson Medical School

675 Hoes Lane West

Piscataway, NJ 08854

Email: davebe97@gmail.com

Tel: 732-235-5394

Fax: 732-235-4990

Derek Adler, BS

Rutgers Molecular Imaging Center Manager

41 Gordon Road, Suite D, Room 365A

Piscataway, NJ 08854

Email: derek.adler@rutgers.edu

Tel: 848- 445-1563

Patrick Sullivan, PhD

Associate Professor

Division of Geriatrics

Department of Medicine

Duke University

Durham VAMC, GRECC

919-636-1689

p.sullivan@duke.edu

Smita Thakker-Varia, PhD

Associate Professor of Neuroscience and Cell Biology

Rutgers Robert Wood Johnson Medical School

675 Hoes Lane West, Research Building 357A

Piscataway, NJ 08854

Email: [varia@rutgers.edu](mailto:varia@rutgers.edu)

Tel: 732-235-5393

Fax: 732-235-4990

Janet Alder, PhD

Associate Professor of Neuroscience and Cell Biology

Rutgers Robert Wood Johnson Medical School

675 Hoes Lane West, Research Building 357A

Piscataway, NJ 08854

Email: janet.alder@rutgers.edu

Tel: 732-235-5392


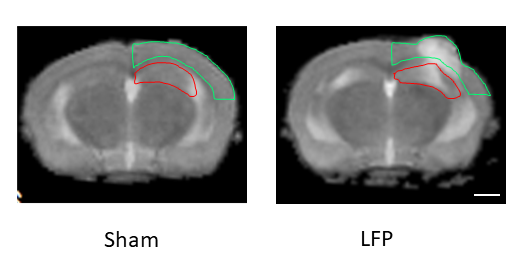


**Supplemental Figure 1.** Representative images of T2 magnetic resonance imaging scans of individual mice after rmTBI or sham procedure. The general area of the cortex where immunohistochemical counts were done is indicated by the green shape. The red area indicates site of the hippocampus, the entire area of which was counted. Scans were conducted at the Rutgers University Molecular Imaging Center with the center’s M2 Compact High-Performance MRI (1T). Scale bars = 3mm.


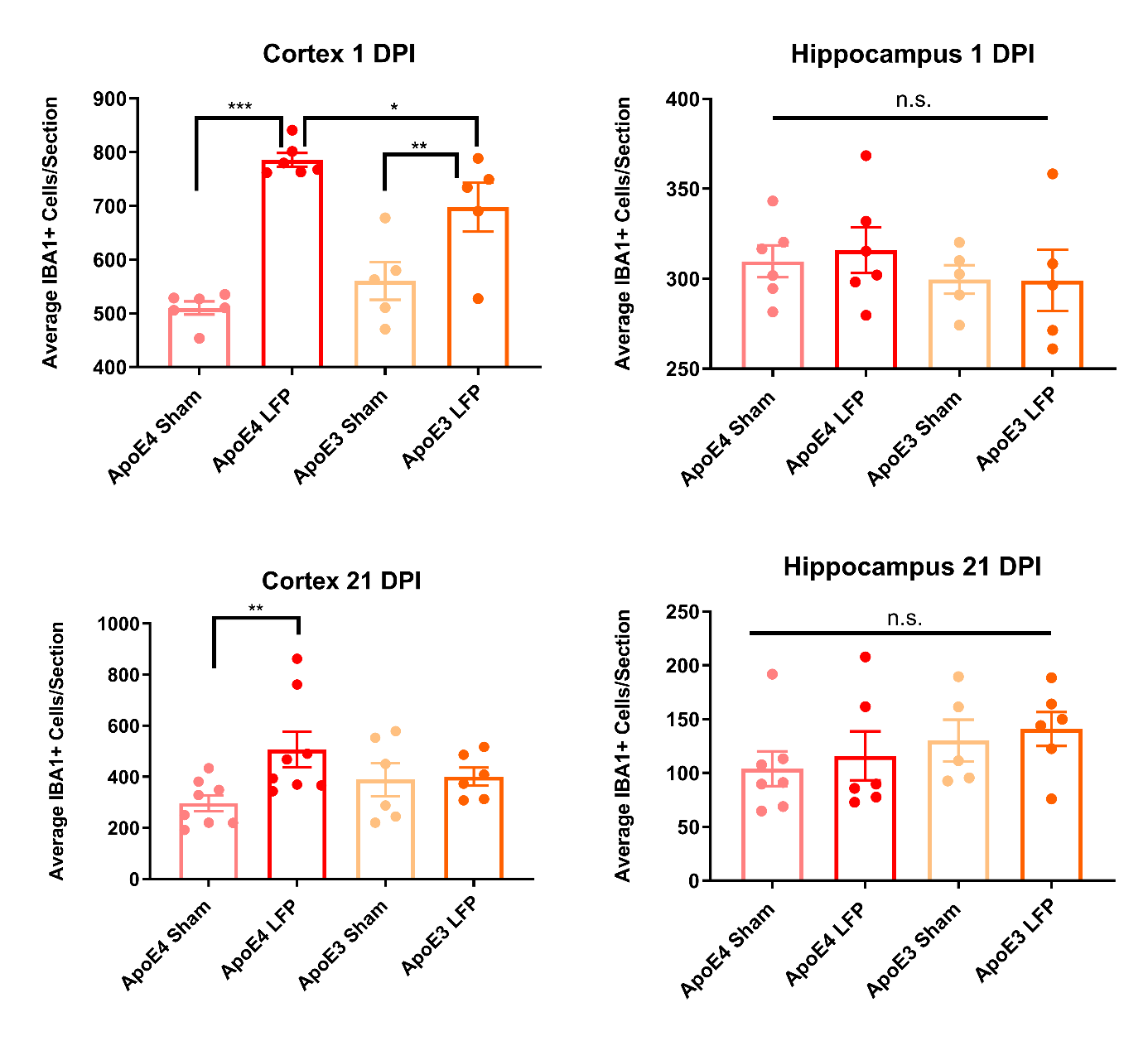


**Supplemental Figure 2.** Repeated mild LFP injury causes an increase in total ionized calcium binding adaptor molecule 1 (IBA1) positive in the brains of injured *APOE4* mice compared to injured *APOE3* mice at 1 DPI in the cortex but not at 21 DPI or in the hippocampus. Quantitation of the average number of total IBA1+ positive cells per cortex and hippocampus ± SEM. * p < 0.05, ** p < 0.01, *** p < 0.001 ANOVA Fisher’s PLSD post-hoc test relative to indicated groups, n =5-8.


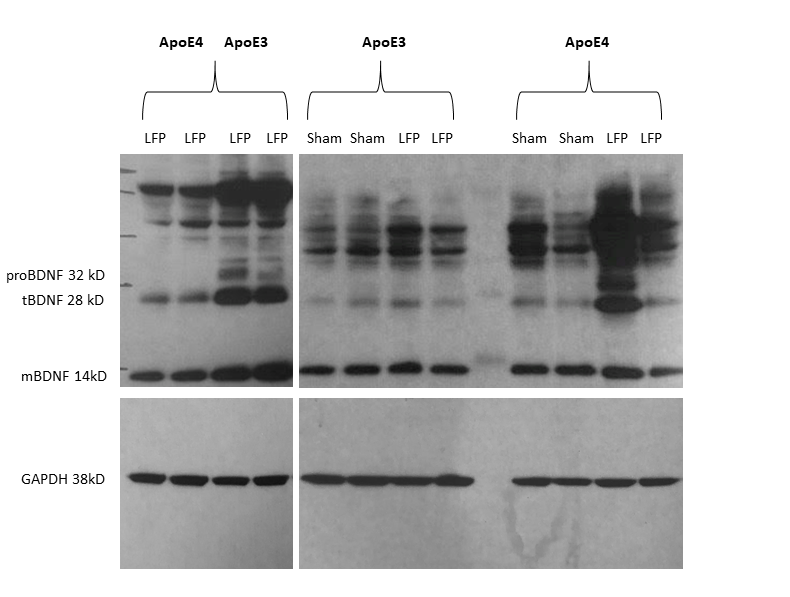


**Supplemental Figure 3**. Repeated mild LFP injury causes an increase in total BDNF expression in *APOE4* mice compared to *APOE3* mice in the cortex and hippocampus at 1 and 21 DPI. Full representative Western Blots showing total BDNF and GAPDH expression after injury from cortical tissue at 21 DPI. In addition to the 32 kD proBDNF, 28 kD tBDNF, and 14kD mBDNF bands, non-specific bands are visible at high molecular weights as has been reported previously for BDNF. Each lane represents one animal.


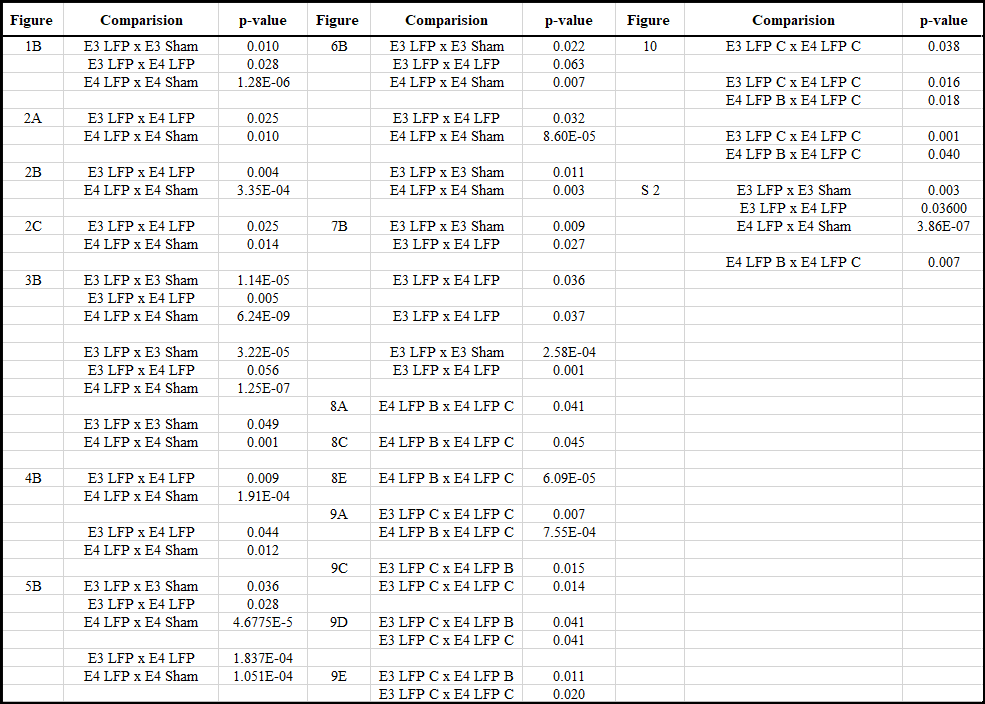


**Supplemental Figure 4.** Graph of the statistical data for the figures. Statistics were carried out on the data sets that were analyzed and the significant differences between groups were noted in the individual figures. This figure provides the p-values for these differences.

**
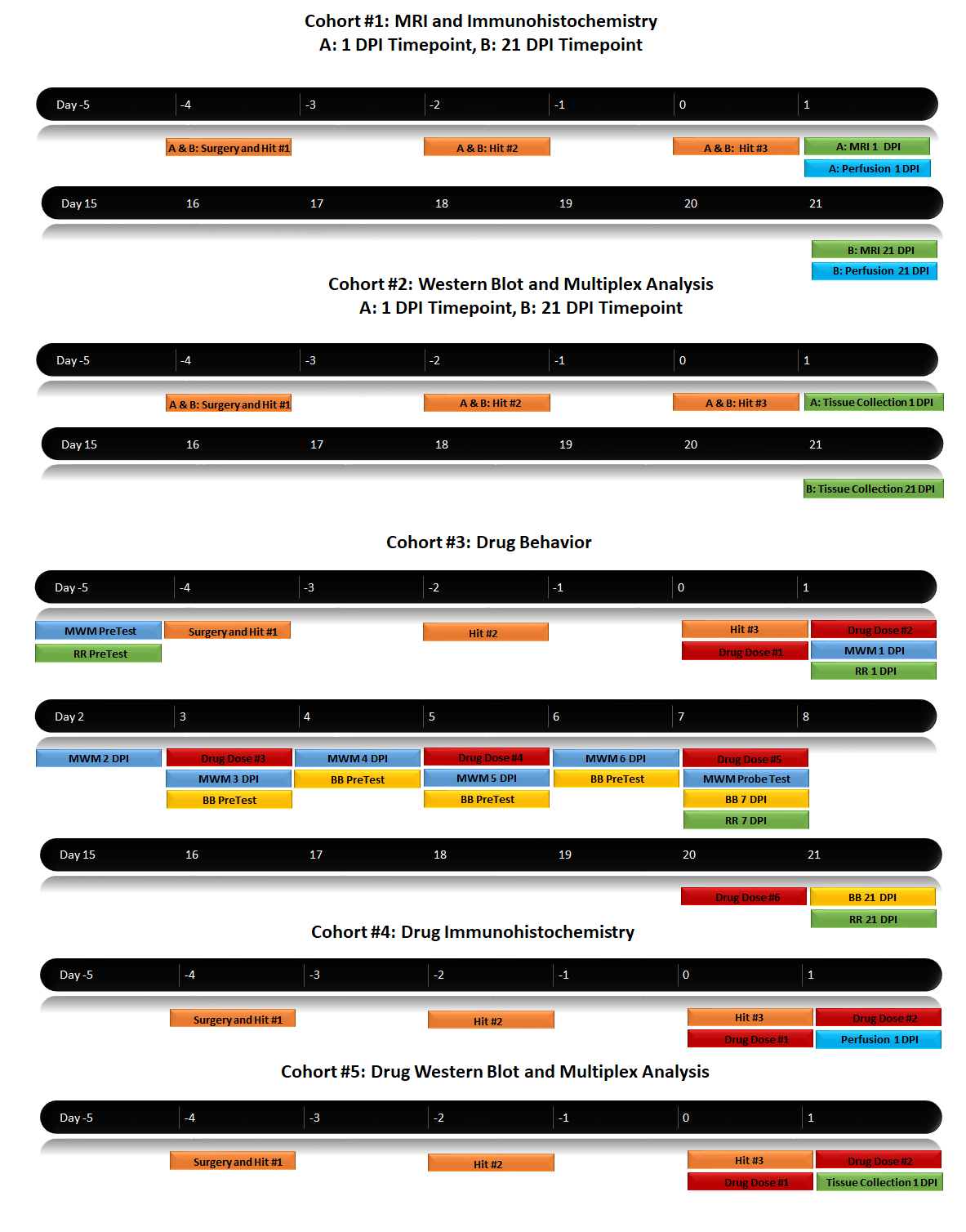
**

**Supplemental Figure 5.** Schematic of the cohort timelines. (Abbreviations are as follows: MRI = Magnetic Resonance Imaging, MWM = Morris Water Maze, RR = Rotarod, BB = Balance Beam.)
